# A novel pH-dependent membrane peptide that binds to EphA2 and inhibits cell migration

**DOI:** 10.7554/eLife.36645

**Published:** 2018-09-17

**Authors:** Daiane S Alves, Justin M Westerfield, Xiaojun Shi, Vanessa P Nguyen, Katherine M Stefanski, Kristen R Booth, Soyeon Kim, Jennifer Morrell-Falvey, Bing-Cheng Wang, Steven M Abel, Adam W Smith, Francisco N Barrera

**Affiliations:** 1 Department of Biochemistry & Cellular and Molecular BiologyUniversity of TennesseeKnoxvilleUnited States; 2 Department of ChemistryUniversity of AkronAkronUnited States; 3 Department of Physiology and BiophysicsCase Western Reserve UniversityClevelandUnited States; 4 PharmacologyCase Western Reserve UniversityClevelandUnited States; 5 Rammelkamp Center for ResearchMetroHealth Medical CenterClevelandUnited States; 6 Graduate School of Genome Science and Technology, University of TennesseeKnoxvilleUnited States; 7 Biosciences DivisionOak Ridge National LaboratoryOak RidgeUnited States; 8 Department of Chemical and Biomolecular EngineeringUniversity of TennesseeKnoxvilleUnited States; 9 National Institute for Mathematical and Biological Synthesis, University of TennesseeKnoxvilleUnited States; Fred Hutchinson Cancer Research CenterUnited States; Institut CurieFrance

**Keywords:** EphA2 activation, receptor tyrosine kinase, membrane active peptide, pH responsive, None

## Abstract

Misregulation of the signaling axis formed by the receptor tyrosine kinase (RTK) EphA2 and its ligand, ephrinA1, causes aberrant cell-cell contacts that contribute to metastasis. Solid tumors are characterized by an acidic extracellular medium. We intend to take advantage of this tumor feature to design new molecules that specifically target tumors. We created a novel pH-dependent transmembrane peptide, TYPE7, by altering the sequence of the transmembrane domain of EphA2. TYPE7 is highly soluble and interacts with the surface of lipid membranes at neutral pH, while acidity triggers transmembrane insertion. TYPE7 binds to endogenous EphA2 and reduces Akt phosphorylation and cell migration as effectively as ephrinA1. Interestingly, we found large differences in juxtamembrane tyrosine phosphorylation and the extent of EphA2 clustering when comparing TYPE7 with activation by ephrinA1. This work shows that it is possible to design new pH-triggered membrane peptides to activate RTK and gain insights on its activation mechanism.

## Introduction

Eph receptors are the largest sub-group of the transmembrane receptor tyrosine kinase (RTK) family (Kania and Klein, 2016; Lemmon and Schlessinger, 2010) and are divided in two classes, EphA and EphB. Humans have nine different EphA and five EphB receptors that are activated, with some exceptions, by ephrinA and ephrinB ligands, respectively (Lisabeth et al., 2013). In general, Eph receptors and ephrin ligands are found on opposing cells, where they establish cell-to-cell contacts (Kania and Klein, 2016; Lisabeth et al., 2013). Full activation of Eph receptors is achieved upon clustering of receptors at the plasma membrane (Kullander and Klein, 2002; Barquilla and Pasquale, 2015; Davis et al., 1994). EphrinA molecules are anchored to the extracellular face of the plasma membrane by a glycosylphosphatidylinositol linkage. Binding of ephrinA ligands to EphA causes cell repulsion through activation of intracellular signaling pathways that control cytoskeletal dynamics. As a result, the EphA-ephrinA signaling axis controls contact-dependent cell communication that drives cell adhesion, migration, morphology, and survival (Kania and Klein, 2016; Boyd et al., 2014). These activities are important during development, particularly in nervous system formation and blood vessel remodeling, and in adult homeostasis of neural, bone and epithelial tissues (Barquilla and Pasquale, 2015).

Not surprisingly, misregulation of EphA-ephrinA signaling can lead to pathological states. For example, it has been found that altered localization of EphA4 contributes to synaptic dysfunction in Alzheimer’s disease (Rosenberger et al., 2014), while a missense mutation in EphA2 can cause age-related cortical cataracts (Barquilla and Pasquale, 2015; Jun et al., 2009). Moreover, Eph receptors can contribute to cancer malignancy. Indeed, Eph receptors were named after their discovery in an erythropoietin-producing hepatoma cell line (Hirai et al., 1987). Relevant to cancer, the EphA2-ephrinA1 signaling axis regulates events crucial for cellular transformation and malignancy. Furthermore, EphA2 is overexpressed in multiple cancer types (breast, brain, ovary, bladder, prostate, pancreas, esophagus, lung, and stomach) (Tandon et al., 2011). However, regulation of EphA2 is complex, and several factors, including ligand binding and downstream events, can cause EphA2 to act as a tumor suppressor or as an oncogenic protein (Shi and Wang, 2018).

EphA2 contains a N-terminal extracellular domain (ECD) connected to the intracellular domain (ICD) by a single transmembrane (TM) helix. Information from ephrinA1 binding to the extracellular ligand-binding domain is transmitted across the membrane by the TM helix in the form of a conformational change in the ICD. As a result, the intracellular kinase domain is activated and auto-phosphorylates multiple tyrosine residues, which triggers a signaling cascade (Binns et al., 2000; Locard-Paulet et al., 2016). Full activation of EphA2 requires first dimerization, a process mediated by the TM domain (Bocharov et al., 2010; Sharonov et al., 2014), as well as soluble domains (Shi et al., 2017; Himanen et al., 2010), followed by assembly into clusters. However, EphA2 activation is poorly understood, especially at the level of the conformational interplay between the TM and soluble domains. New tools are needed to interrogate the conformational rearrangements that mediate EphA2 activation.

We have recently designed the ATRAM (acidity-triggered rational membrane) peptide. ATRAM is a highly soluble synthetic peptide that is capable of pH-dependent interaction with lipid membranes: at neutral pH, ATRAM binds to the membrane surface, while a decrease in pH triggers insertion into the lipid bilayer as a TM helix (Nguyen et al., 2015). The pH-dependent membrane insertion of ATRAM results from the protonation of glutamic acid residues, as this event switches the polarity of the peptide from moderately to highly hydrophobic. The pH-triggered membrane insertion of ATRAM and similar peptides can be used to target cell membranes in acidic environments (Reshetnyak et al., 2007). Acidosis of the extracellular medium is a hallmark of aggressive tumors and results from altered cell metabolism and physiology (Schornack and Gillies, 2003). Tumor acidosis favors aggressiveness, metastasis and invasion (Martinez-Outschoorn et al., 2011). We reasoned that the strategy used to design ATRAM could be applied to conditionally solubilize the transmembrane domain of a receptor. Here, we have used this approach to transform the TM helix of the human EphA2 into an amphitropic peptide, called TYPE7 (transmembrane tyrosine kinase peptide for Eph). TYPE7 is a highly soluble peptide in aqueous solution that inserts into cellular membranes in a pH-dependent fashion. The TM state of TYPE7 interacts with EphA2 to induce receptor oligomerization and phosphorylation, which causes inhibition of cell migration. The observed mechanistic differences between EphA2 activation by eprhinA1 and TYPE7 provide new insights into the activation mechanism of EphA2.

## Results

### A pH decrease triggers the membrane insertion of TYPE7

**Figure 1.**
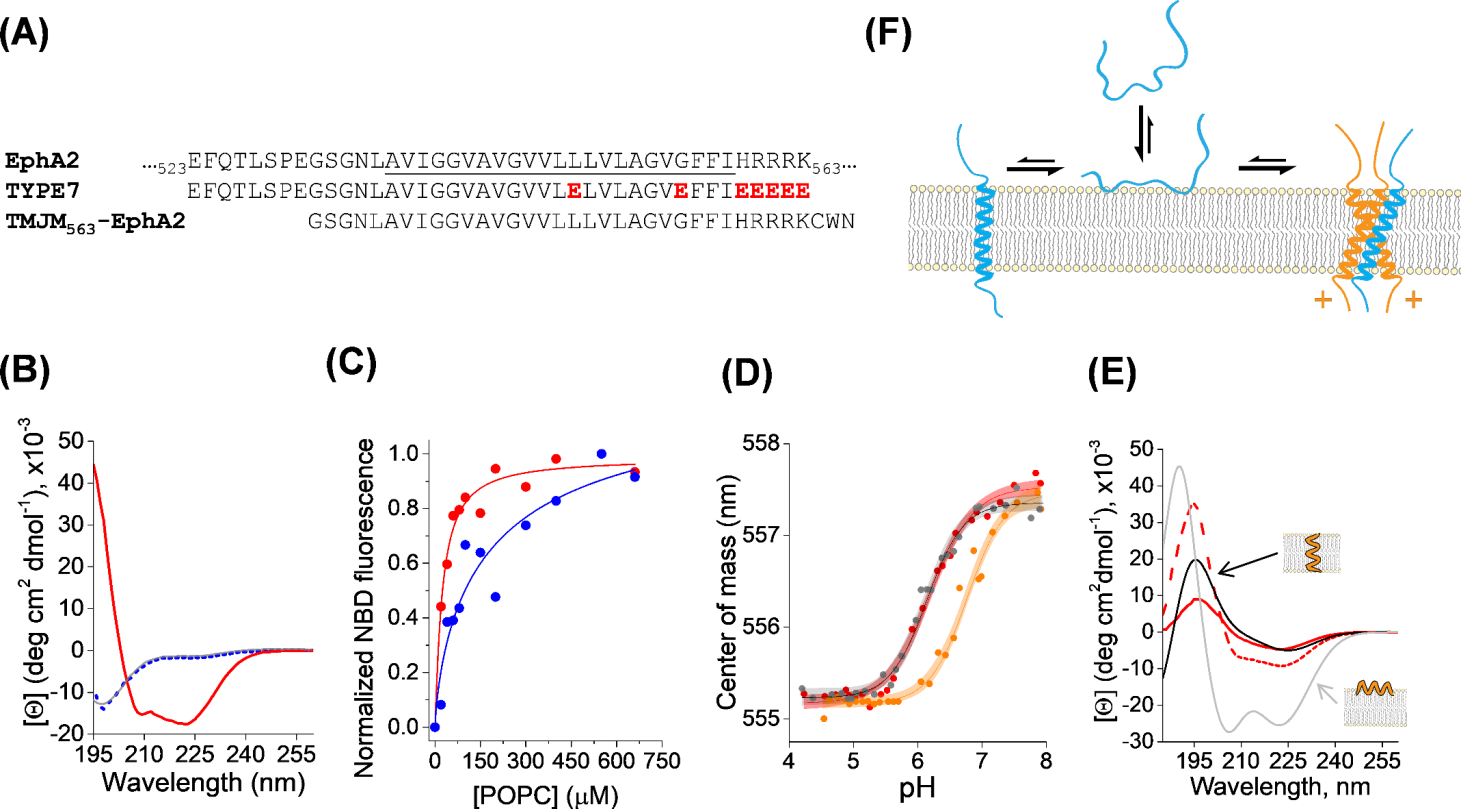
Membrane interaction of TYPE7. (**A**)* Top,* partial amino acid sequence of the human EphA2 receptor showing the TM helix (underlined), preceded by a short extracellular segment, and followed by the start of the juxtamembrane segment. Residue numbers in the sequence of EphA2 are shown. *Middle*, sequence of the TYPE7 peptide, where the acidic residues introduced are shown in red. *Bottom*, sequence of the TMJM_563_-EphA2 peptide used in panel D. (**B**) Circular dichroism determination of TYPE7 secondary structure in buffer at pH 8 (grey line), and in the presence of POPC vesicles at pH 8 (dotted blue line) and after acidification to pH 4 (red line). (**C**) TYPE7 binding to POPC vesicles at pH 5 (red) and pH 8 (blue). Lines are fittings to Equation. 3, used to determine the *Kp* values. Lipid binding was measured using the environmentally-sensitive dye NBD attached to the N_t_ of TYPE7. (**D**) Determination of the pH midpoint (pH_50_) for the insertion of TYPE7 into POPC vesicles. TYPE7 data is shown in red symbols. Data obtained in vesicles containing the GWALP23 peptide control are shown in grey, and in vesicles containing TMJM_563_-EphA2 in orange. Peptide insertion was monitored by following changes in the NBD spectral center of mass (Equation. 1) (Scott et al., 2017; Barrera et al., 2002). Control OCD experiments showed that TMJM_563_-EphA2 formed a TM helix (Figure 1—figure supplement 4). The lines correspond to the fitting to the data using Equation. 2 and 95% confidence intervals are shown as shaded areas (*n* = 6). (**E**) OCD determination of the membrane orientation of TYPE7. Data were obtained in POPC (16:0,18:1-PC, dashed red line) and 22:1,22:1-PC (continuous red line). The theoretical spectra for a perfectly transmembrane (0°, black line) and peripheral (90°, grey line) helix are shown as a reference. (**F**) Cartoon of the different states TYPE7 (blue) adopts, and how TMJM_563_-EphA2 (orange) promotes the TM state of TYPE7. Arrows represent approximate equilibrium conditions found at pH ~6.5. The (+) symbols represent basic residues in the juxtamembrane segment of EphA2.

TYPE7 is comprised of the sequence of the TM region of EphA2 and flanking residues (Figure 1A). We introduced five glutamic acid residues at the C-terminus and two in the TM region to enhance water solubility and confer pH-responsiveness. TYPE7 dissolved readily in buffer (Figure 1—figure supplement 1), and circular dichroism (CD) spectroscopy experiments showed that TYPE7 was unstructured in solution at neutral and slightly basic pH (as indicated by the single minimum at ~200 nm, grey line in Figure 1B and Figure 1—figure supplement 1). However, in the presence of phosphatidylcholine (POPC) lipid vesicles, TYPE7 bound to lipids (Figure 1B,C), without causing bilayer disruption (Figure 1—figure supplement 2). We used a NBD dye as a reporter for lipid interaction, and observed that the TYPE7-NBD lipid affinity was pH-dependent. While the lipid partition coefficient (K*p*) at pH 8 was 0.8 × 10^6^ (±0.4×10^6^), at pH 5 it increased to 2.9 × 10^6^ (±0.4×10^6^) (mean ± S.E.M, *n* = 3). This result is in agreement with our expectation of TYPE7 being more hydrophobic at acidic pH, as a result of side chain protonation of glutamic acids. Next, we performed a complete pH titration in the presence of POPC vesicles, and observed that TYPE7-NBD fluorescence changed in a sigmoidal fashion (Figure 1D, red line), with a pH midpoint (pH_50_) of 6.18. We used CD to determine the conformation that TYPE7 adopts in the presence of lipids at neutral and acidic pH. At close to neutral pH TYPE7 was unfolded (see Figure 1B, dotted blue line), while at acidic pH the two characteristic α-helical minima were observed. This change indicates that the pH titration involves membrane helical formation.

**Figure 1—figure supplement 1.**
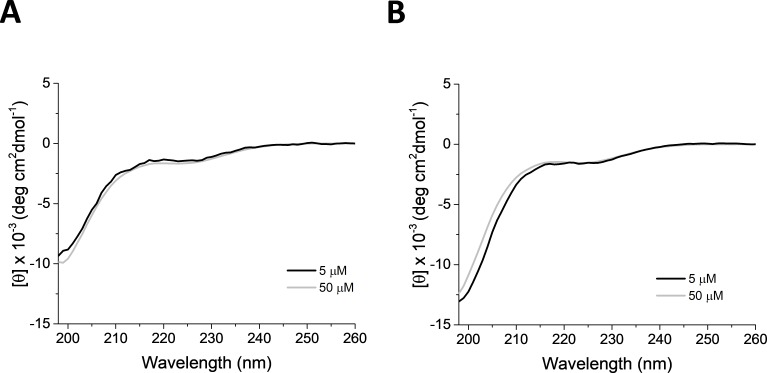
TYPE7 solubility. Circular dichroism (CD) solubility studies of TYPE7 in PBS (11.9 mM NaPi, 137 mM NaCl, 2.7 mM KCl, pH 7.4) (**A**) and 10 mM NaPi pH 8 (**B**) solutions. Normalized CD spectra show a single minimum at 200 nm. The lack of significant secondary structure suggests that the peptide is not aggregated at the two concentrations tested, 5 μM and 50 μM (black and grey lines, respectively); *n* = 3.

**Figure 1—figure supplement 2.**
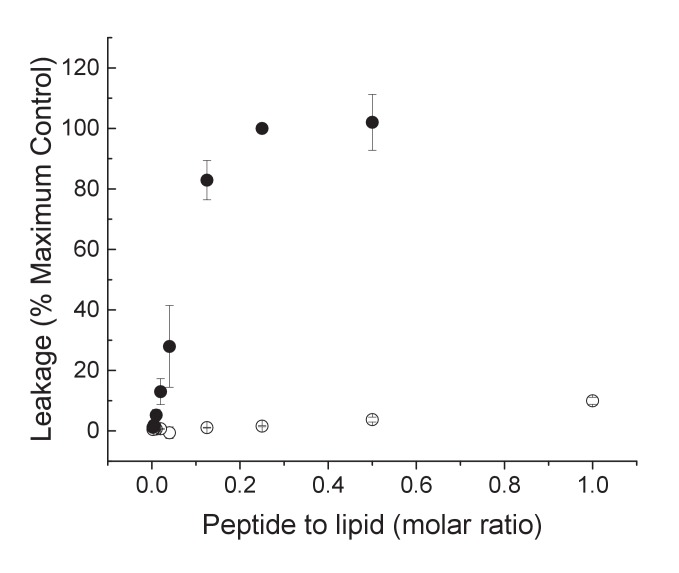
TYPE7 does not induce significant membrane leakage. The release of calcein encapsulated in POPC vesicles was measured by following the fluorescence intensity at 485 nm after addition of TYPE7 (open symbols). For a constant POPC concentration, different peptide concentrations were tested, for a 0.0025 – 1.0 mol % TYPE7:POPC molar ratio range. Mellitin was used as a positive control for leakage (closed symbols). Maximum leakage was achieved by addition of Triton X-100. Mean ±S.D., *n* = 3.

**Figure 1—figure supplement 3.**
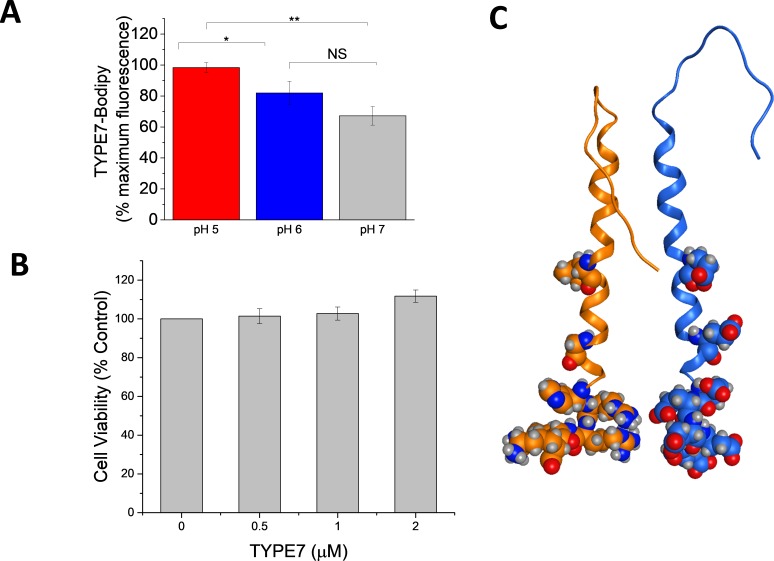
TYPE7 is not toxic, and shows a pH-dependent interaction with cells. (**A**) TYPE7-bodipy FL-X binding to H358 cells at pH 5, 6 and 7. Data at different pH values were normalized to maximum fluorescence. Mean ±S.D., *n* = 3. Student’s *t*-test; *p<0.05; **p<0.01 and NS: not significant. (**B**) H358 cells were treated with increasing concentrations of TYPE7 (0.5, 1 and 2 μM) during 24 hr. Cell viability was assessed using the MTS assay. The results indicate that TYPE7 does not cause toxicity to treated cells. Mean ±S.D., *n* = 3. (**C**) We threaded the sequence of TYPE7 (blue) onto one of the helices of the published dimeric structure of the transmembrane domain of EphA2 (PDB: 2K9Y) (orange). The residues substituted with glutamic acid are shown as spheres on TYPE7 outside the helix interface. The corresponding EphA2 residues are highlighted on the opposite orange helix.

**Figure 1—figure supplement 4.**
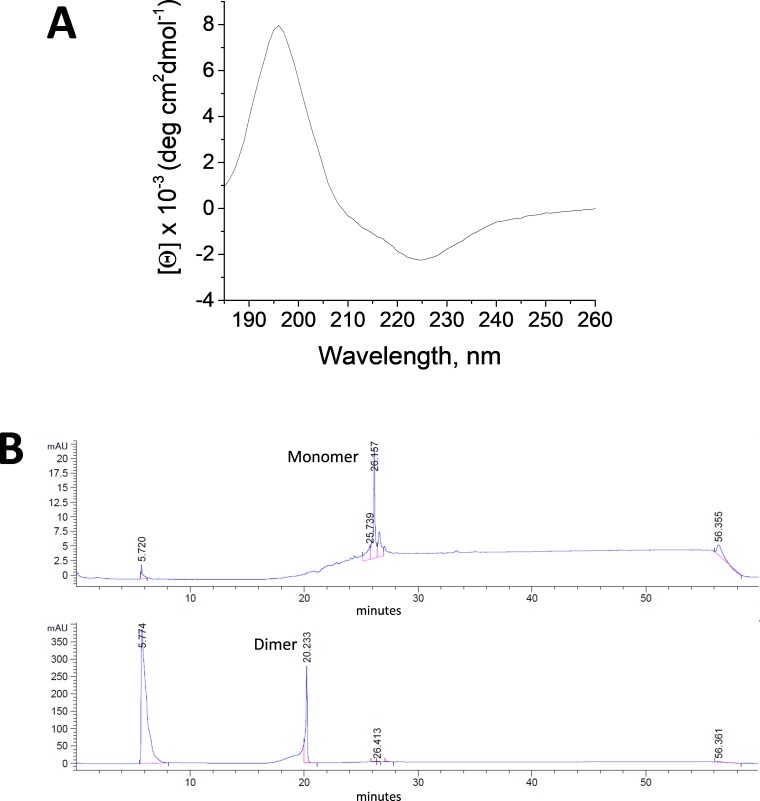
TM-EphA2 peptide inserts into membranes as a transmembrane helix. (**A**) OCD spectrum of TMJM_563_-EphA2 in POPC (16:0,18:1-PC) bilayers. (**B**) HPLC data showing that TMJM_563_-EphA2 does not dimerize using a disulfide bond. *Top*, chromatogram showing the elution of the TMJM_563_-EphA2 monomer at 26.2 min. *Bottom*, control experiment where TMJM_563_-EphA2 dimerization was induced by oxidation with 10 mM copper phenanthroline for 3.5 hr. A dimeric peak appears at 20.2 min, which was not observed in the absence of oxidizing agent.

Oriented circular dichroism (OCD) can determine the alignment of an α-helix with respect to the plane of hydrated supported bilayers. Figure 1E depicts the theoretical OCD spectra corresponding to a TM α-helix lying on the membrane surface (grey line) and inserted into the membrane (black line), where the 208 nm helical minimum is almost absent (Wu et al., 1990; Bürck et al., 2016; Ulmschneider et al., 2014). We used OCD to determine the helical membrane orientation that TYPE7 adopts in POPC at pH 5. We observed that the OCD spectrum (Figure 1E, dashed red line) was closer to the theoretical curve for a TM helix. However, the differences with the black line suggest that TYPE7 adopted a tilted TM helix orientation in POPC bilayers. Transmembrane peptides will typically tilt in the membrane to avoid hydrophobic mismatch (Killian and Nyholm, 2006). We reasoned that if TYPE7 formed a TM helix, its membrane tilt would decrease to adapt to a thicker lipid membrane (Anbazhagan and Schneider, 2010). To test this idea, we repeated the OCD experiment in 22:1,22:1-PC, a lipid with longer acyl chains that forms bilayers 7.3 Å thicker than POPC (16:0,18:1-PC) (Kucerka et al., 1808; Kucerka et al., 2009). In agreement with the expected behavior for a TM helix, the OCD spectrum in the thicker bilayer was closer to the theoretical TM curve with no tilt. Taken together, our data reveal that the sigmoidal pH titration (Figure 1D), associated with increased lipid affinity (Figure 1C), represents the transition from an unstructured state bound to the membrane surface to a transmembrane helix found at lower pH (Figure 1F).

### TYPE7 shows no toxicity and binds to cells in a pH-dependent manner

We explored if TYPE7 was also able to bind to cellular membranes. To this end, we studied the cellular interaction of a fluorescently labeled version of TYPE7 at different pH values (Figure 1—figure supplement 3A). We observed a robust interaction with cells at neutral pH, which increased with acidification. This indicates that the enhanced lipid affinity at acidic pH values is observed both in lipid vesicles and in cells. However, since satisfactory TYPE7 cell binding was achieved at neutral pH, we decided to employ physiological pH for the ensuing cellular experiments. Additionally, we performed cell viability experiments to study if TYPE7 was toxic to cells. The results of an MTS assay indicated that the peptide did not decrease cell viability (Figure 1—figure supplement 3B).

### TYPE7 interacts with EphA2

Next, we investigated if TYPE7 could interact with EphA2. The single TM helix of EphA2 forms a dimer that mediates receptor dimerization (Bocharov et al., 2010; Sharonov et al., 2014; Singh et al., 2015). The TM region of TYPE7 contains glutamic acid residues designed to align into a single helical face. NMR studies indicated that this face is located away from the dimerization interface of EphA2 (Figure 1—figure supplement 3C) (Bocharov et al., 2010). As a result, TYPE7 theoretically contains an intact dimerization interface to interact with the EphA2 TM helix. We hypothesized that this would allow binding of TYPE7 to the transmembrane domain of EphA2. To evaluate this hypothesis, we used a new peptide encompassing the TM domain of EphA2 and five residues at the N-terminus of the juxtamembrane segment (JMS), through residue K563 (Figure 1A). We refer to this peptide as TMJM_563_-EphA2 (Figure 1—figure supplement 4). We used the pH_50_ assay to study the interaction between TYPE7 and TMJM_563_-EphA2. We reasoned that transmembrane binding between TMJM_563_-EphA2 and TYPE7 would increase the pH_50_, as the TM state of TYPE7 would be stabilized over the surface-bound conformation, displacing the equilibrium (Figure 1F). Indeed, the presence in the vesicles of TMJM_563_-EphA2 at a 4-fold molar excess, increased the pH_50_ of TYPE7 from 6.18 ± 0.09 to 6.85 ± 0.16 (mean ±S.D., *n* = 7 – 9). Interestingly, Figure 1D shows that, in these conditions, a non-negligible fraction of TYPE7 is already in the TM state at pH 7. This suggests the intriguing possibility that TYPE7 could interact with EphA2 without requiring strong acidification. To study the specificity of this effect we performed a control experiment replacing TMJM_563_-EphA2 by GWALP23, an unrelated peptide that also forms a transmembrane helix (Ozdirekcan et al., 2005; Holt et al., 2009; Rankenberg et al., 2012). The pH_50_ of TYPE7 was similar in the absence or presence of GWALP23 (6.18 ± 0.09 and 6.17 ± 0.20, respectively, Figure 1D), suggesting that TMJM_563_-EphA2 specifically interacts with TYPE7.

**Figure 2.**
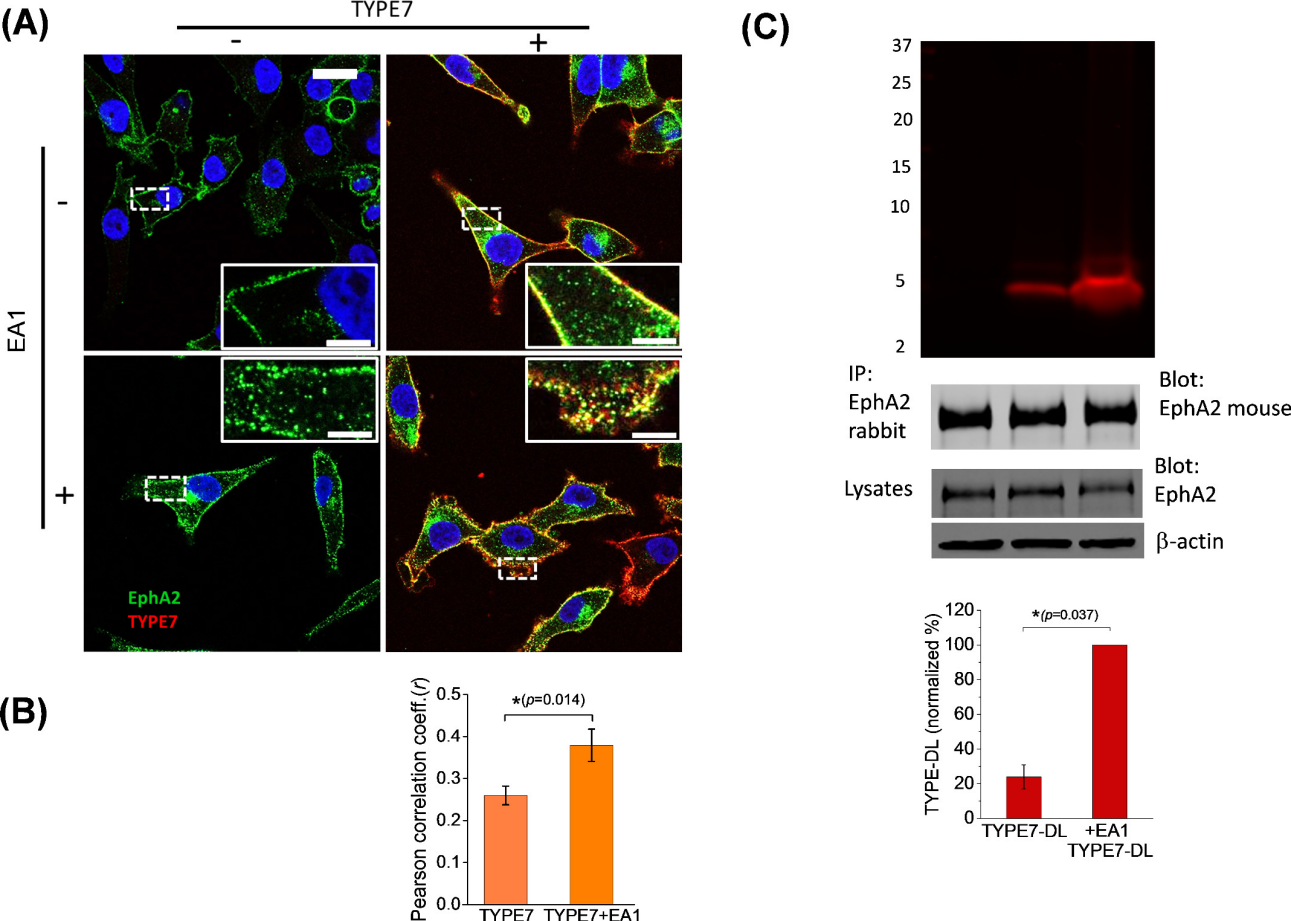
TYPE7 interacts with endogenous EphA2 in cells. (**A**) Confocal microscopy shows co-localization of TYPE7 and EphA2. A375 cells were incubated in the presence (+) or absence (-) of 0.5 µg/mL EA1 and 0.2 µM TYPE7-Alexa568 (red) for 5 min at room temperature. Cells were fixed and endogenous EphA2 was labeled via immunofluorescence (green). Images were collected using a 63x objective, and insets show images corresponding to the white dashed areas collected with a 100x objective. Scale bars are 20 µm and 5 µm, respectively. (**B**) The Pearson correlation coefficient (r) was calculated for cells incubated with TYPE7 in the absence and presence of EA1. Bar graph shows mean ±S.D. Student’s *t*-test was performed for 14 – 17 images. *p<0.05, with as effect size of 0.80 standard deviations, *n* = 2. (**C**) *Top,* SDS-PAGE showing that TYPE7-DL co-precipitates with endogenous EphA2 when using a polyclonal anti-rabbit EphA2 antibody. *Middle*, control Western blots of EphA2 immunoprecipitation blotted with mouse anti-EphA2 show that similar amounts of endogenous EphA2 were pulled down in all samples. Total cell lysates blotted with EphA2 and β-actin indicate that similar levels of protein were loaded. *Bottom,* quantification of the fluorescent bands. Bar graph shows mean ±S.D. as a percentage of maximum intensity. A Mann-Whitney test was performed (*p<0.05), *n* = 3.

To explore the cellular relevance of the biophysical results, we examined the co-localization of TYPE7 with endogenous EphA2 in A375 cells at physiological pH. We evaluated the effect of EphA2 activation on the interaction between TYPE7 and EphA2 by treating the cells with ephrinA1-Fc (EA1). EA1 uses a Fc group (heavy chain of human IgG1) to crosslink ephrinA1. Incubation with EA1 recapitulates EphA2 trans-activation by membrane clusters of ephrinA1 (Davis et al., 1994). The resulting EphA2 clustering and phosphorylation leads to recycling into endosomes and degradation (Kania and Klein, 2016; Nikolov et al., 2014; Boissier et al., 2013; Sabet et al., 2015). We used confocal microscopy to study the cellular distribution of EphA2. As expected, we observed that EA1 promoted EphA2 clustering, resulting in accumulation of large puncta at the plasma membrane and cytosolic recycling (compare two insets in left column of Figure 2A). We used TYPE7 fluorescently labelled with Alexa568 to assess co-localization with EphA2. We observed that the TYPE7 signal overlapped to a large degree with the EphA2 receptor in the plasma membrane (Figure 2A, upper right panel). However, this could partially result from the membrane affinity of TYPE7 (Figure 1C). To test the specificity of the co-localization, we performed additional experiments in the presence of EA1, and evaluated if TYPE7 partitioned to the clusters. Interestingly, after EA1 activation we observed stronger TYPE7 co-localization with EphA2 (Figure 2A, lower right panel). We quantified co-localization using the Pearson correlation coefficient (*r*) (Figure 2B) (Manders et al., 1992), which showed that the positive pixel correlation between EphA2 and TYPE7 (*r* = 0.26, *n* = 14) increased significantly upon receptor activation with EA1 (*r* = 0.38, *n* = 17) (*t* = −2.68, p<0.05). Next, we performed a co-precipitation assay to confirm the interaction between TYPE7 and endogenous EphA2. To this end, we treated H358 cells with TYPE7 labelled with a near-IR fluorophore, DyLight 680 (TYPE7-DL). After EphA2 immuno-precipitation, SDS-PAGE gels showed a fluorescent band corresponding to the molecular weight of TYPE7-DL (5.2 KDa) (Figure 2C). Interestingly, when EphA2 was activated with EA1, the amount of TYPE7 that precipitated with endogenous EphA2 increased fourfold. These data suggest that the peptide might be trapped in EphA2 clusters. Taken together, the co-localization and co-precipitation results indicate that TYPE7 interacts with EphA2 in cells, and binding is enhanced upon activation of EphA2.

### TYPE7 inhibits cell migration by specific EphA2 phosphorylation at Y772 and decreases Akt phosphorylation

**Figure 3.**
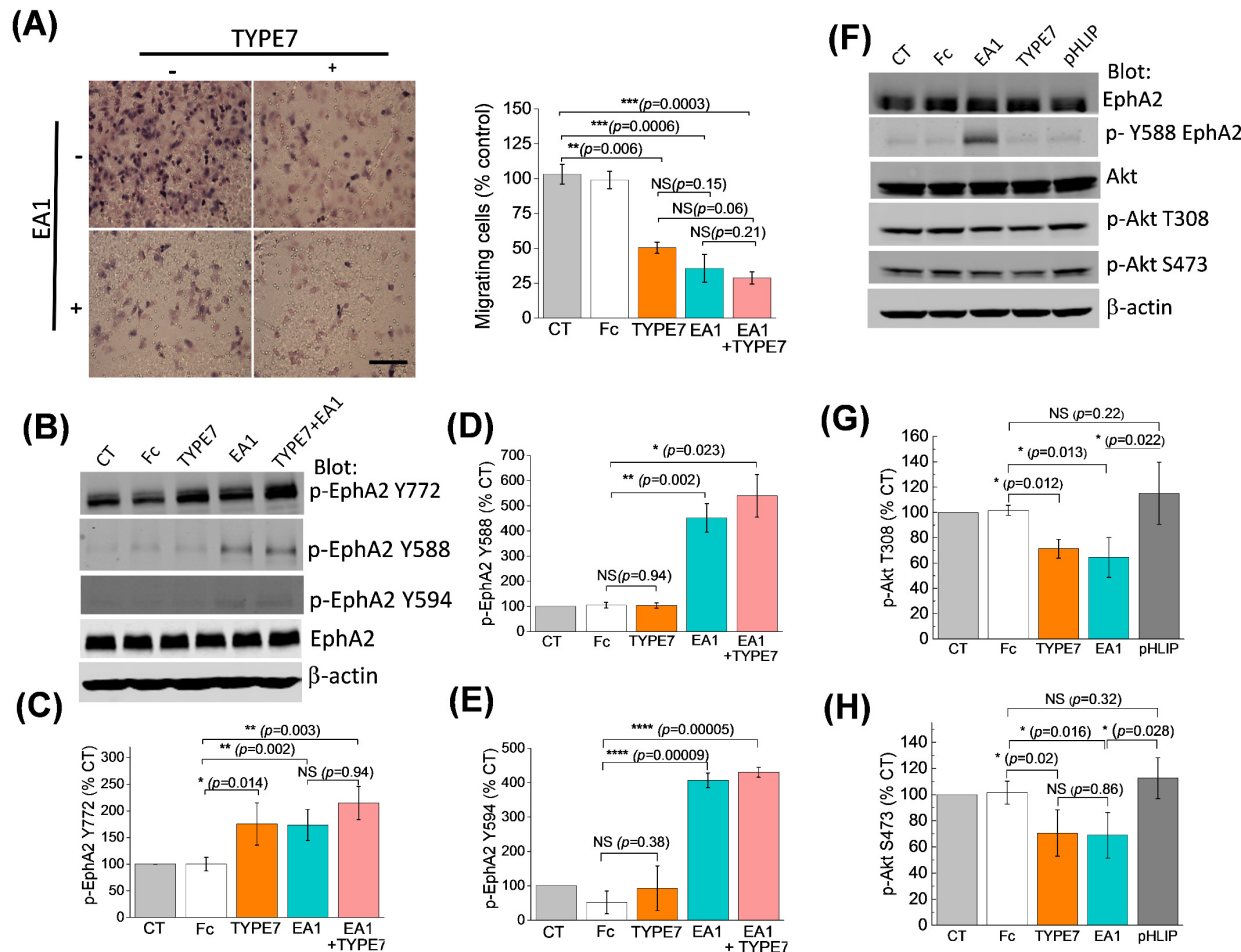
TYPE7 decreases cell migration, and induces EphA2 phosphorylation at Y772 and Akt de-phosphorylation. (**A**) *Left*, cell migration was measured in the presence and absence of TYPE7 and EA1 using a Boyden cell chamber assay. Representative images are shown. *Right*, quantification of migrating cells, showing that incubation with TYPE7 reduced A375 cell migration to a similar degree as EA1, with effect sizes of 8.4 and 12.6 standard deviations from control, respectively. *N* = 3. Cells were treated with an isolated Fc group as a control for the Fc present in EA1. Scale bar is 200 µm (**B–E**), Phosphorylation of Y772 and JMS phosphorylation at Y588 and Y594. A representative Western blot is shown (**B**). Band intensity was quantified for p-Y772 (**C**), p-Y588 (**D**), and p-Y594 (**E**). We found that incubation with TYPE7 increased phosphorylation of Y772 as efficiently as EA1, with effect sizes of 5.1 and 7.7 standard deviations from control, respectively. Mean ±S.D. are shown. *n* = 5. (**F–H**), Phosphorylation levels of Akt. A representative Western blot is shown (**F**) and band intensity was quantified for p-T308 (**G**) and p-S473 (**H**). Lysates were blotted against total EphA2 to detect total protein levels, and β-actin as a loading control. Student’s *t*-test was performed to obtain *p* values (*p<0.05; **p<0.01; ***p<0.001; ****p<0.0001 and NS, not significant).

Next, we determined the functional significance of TYPE7 binding to EphA2. EphA2 controls cell-cell contact, and EphA2 activation inhibits cell migration. The effect of TYPE7 on EphA2-mediated cell migration was tested by using a Boyden chamber assay. EA1 was used as a positive control of ligand-induced inhibition of cell migration (Miao et al., 2000). Figure 3A shows that incubation with TYPE7 reduced A375 cell migration to a similar degree as EA1. Co-incubation of TYPE7 with EA1 did not further inhibit cell migration, indicating that a maximum inhibitory effect had already been obtained with saturating levels of EA1. When we repeated the Boyden chamber assay in H358 cells, we observed that TYPE7 also efficiently inhibited cell migration in this cell type (Figure 3—figure supplement 1).

**Figure 3—figure supplement 1.**
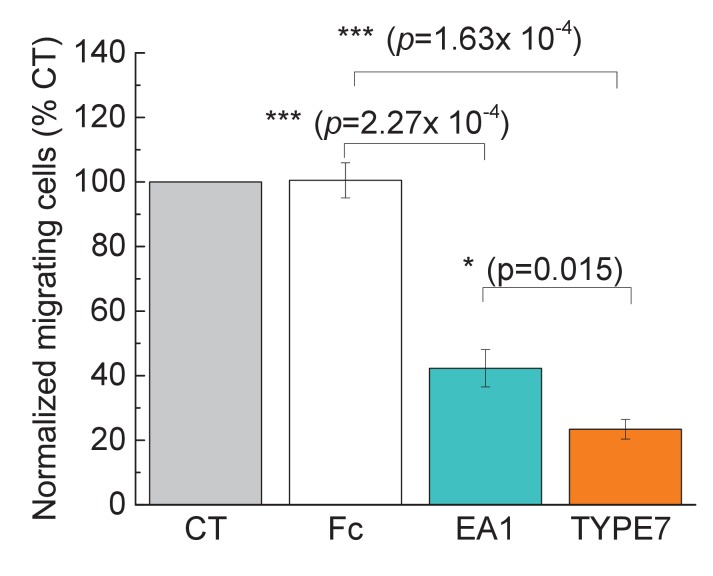
TYPE7 decreases cell migration in H358 cells. Cell migration was measured in the presence and absence of TYPE7 and EA1 using a Boyden cell chamber assay, and the number of migrating cells was normalized to control conditions (CT). The experiment was performed with cells treated with 1 μg/mL Fc, 1 μg/mL EA1, or 2 μM of pHLIP or TYPE7. Statistical analysis was performed by using a Student’s *t*-test; *p<0.05, ***p<0.001. *n* = 3.

**Figure 3—figure supplement 2.**
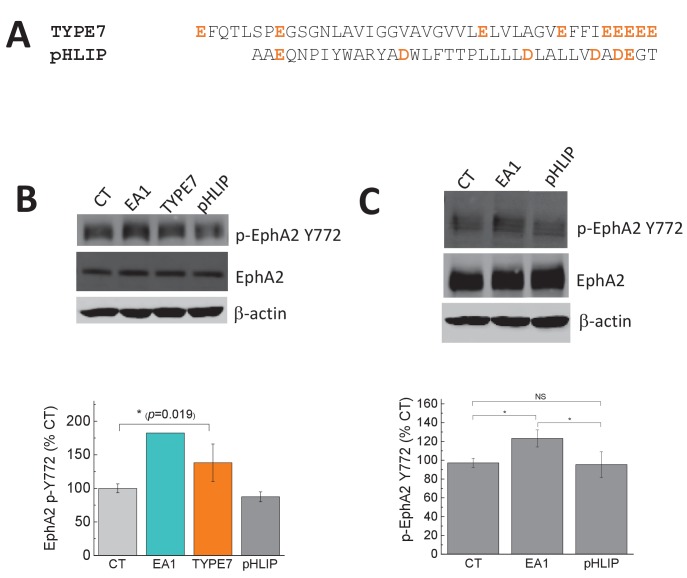
The control pHLIP peptide does not affect the phosphorylation of EphA2 at Y772. (**A**) Comparison of the sequences of TYPE7 and pHLIP, with acidic residues marked in orange. Experiments were performed in A375 cells (**B**) and H358 cells (**C**). *Top panels*, cell lysates were blotted with anti-phospho-EphA2 Y772, and EphA2 and anti-β-actin as loading controls; *Bottom panels*, quantification of p-EphA2 Y772 bands. Cells were treated with Fc, TYPE7 (2 μM), pHLIP (2 μM), or EA1 (0.5 μg/mL). Statistical analysis was performed using a Student’s *t*-test; *p<0.05, NS = no significant differences. *n* = 4 – 6 for panel B, and *n* = 3 for panel C. All experiments were performed at pH 7.4, except pHLIP in panel B, which was performed at pH 4.2 to ensure complete TM helix formation of pHLIP.

**Figure 3—figure supplement 3.**
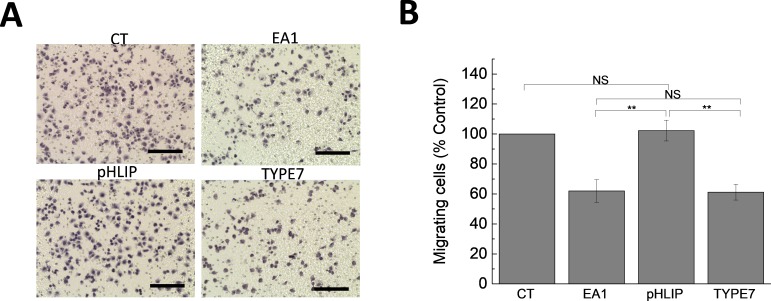
The control pHLIP peptide does not affect cell migration. (**A**) Boyden chamber assay was performed with A375 cells treated with 1 μg/mL Fc (CT), 1 μg/mL EA1, 2 μM pHLIP or 2 μM TYPE7. Scale bars are 200 μm. (**B**) Image quantification. Cells treated with pHLIP migrated similarly as CT. On the contrary, as shown in Figure 3A, TYPE7 and EA1 induced similar levels of cell migration inhibition. Statistical analysis was performed by using a Student’s *t-*test. **p<0.01, NS = no significant differences. *n* = 3.

**Figure 3—figure supplement 4.**
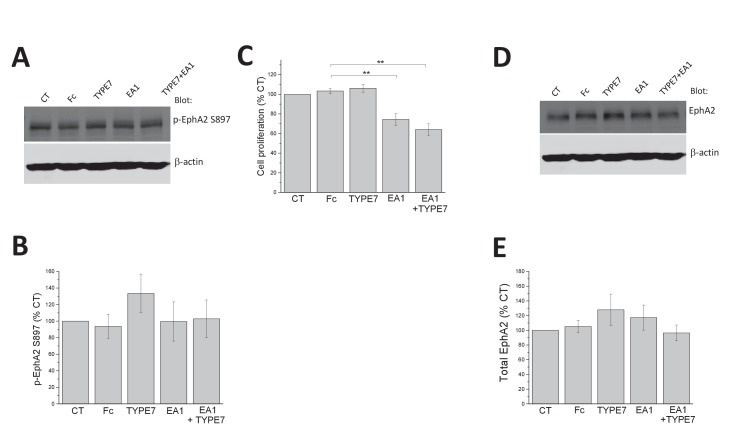
EphA2 expression levels and phosphorylation at S897 are not affected by TYPE7. (**A**) H358 cells were treated with Fc (0.5 μg/mL), TYPE7 (2 μM) or EA1 (0.5 μg/mL). The cell lysates were blotted with anti-phospho-EphA2 S897 and anti-β-actin to assess total protein loading. (**B**) EphA2-phospho-S897 quantification of five independent experiments. Statistical analysis was performed by using a Student’s *t*-test, which indicated no significant differences between samples and controls. (**C**) MTS cell proliferation assay. A375 cells were treated with Fc (0.5 μg/mL), EA1 (3 μg/mL), TYPE7 (2 μM) and TYPE7 +EA1 for 48 hr. No significant differences between Fc control and TYPE7 treated cells were found using a Student’s *t*-test; **p<0.01. Mean ±S.D., *n* = 3. (**D–E**) EphA2 expression levels do not change after TYPE7 treatment. Student’s *t*-test was performed and no significant differences were found between samples. Mean ±S.D., *n* = 5.

**Figure 3—figure supplement 5.**
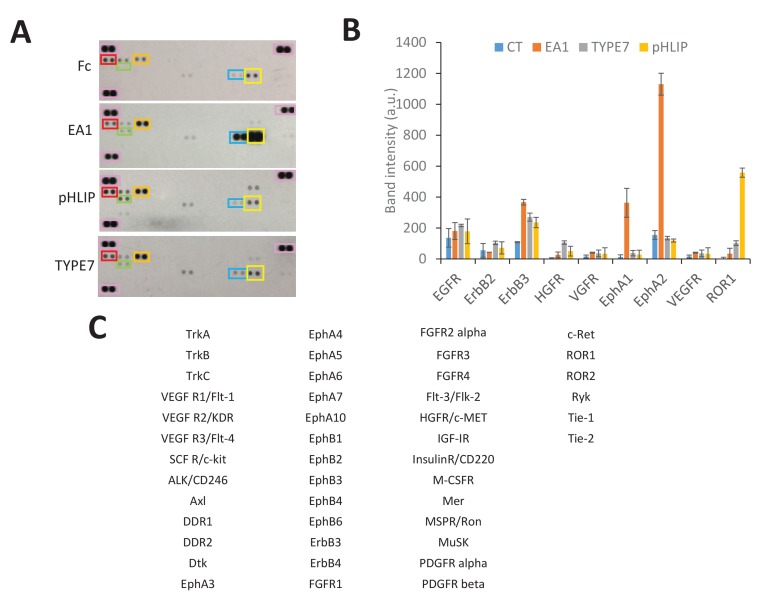
Human Phospho-Tyrosine RTK array. (**A**) H358 cells were treated with Fc (0.5 mg/mL), TYPE7 (2 μM), pHLIP (2 μM) or EA1 (0.5 mg/mL). After treatment, cell lysates were incubated overnight with array membranes to detect tyrosine phosphorylation of 49 different RTKs. The three pairs of reference spots used for blot alignment are boxed pink. Boxed RTK are: EphA1 (blue), EphA2 (yellow), HGFR/c-MET (green), EGFR (red) and ErbB3 (orange). (**B**) Bar graph shows mean and standard deviation of selected RTKs. The table on the right shows the identity of all the RTKs. (**C**) pHLIP weakly promotes phosphorylation of ErbB3 and HGFR/c-MET, as TYPE7 does. Since pHLIP does not induce EphA2 phosphorylation at Y772 (Figure 3—figure supplement 2) or affects cell migration (Figure 3—figure supplement 3), this evidence logically argues against activation of those RTKs being involved in the TYPE7 regulation of these events.

Activation of EphA2 by EA1 causes phosphorylation of tyrosine residues in the juxtamembrane segment (JMS) of the ICD (Y588 and Y594) and the kinase domain activation loop (Y772). Phosphorylation of these residues is followed by a signaling cascade that inhibits cell migration and invasion (Locard-Paulet et al., 2016). To understand TYPE7 anti-migratory effects, we performed Western blots using EphA2 phospho-specific antibodies in H358 cells. We found that incubation with TYPE7 increased phosphorylation of Y772 as efficiently as EA1 (Figure 3B,C). Y772 is located in the activation loop of the kinase domain (Fang et al., 2008; Balasubramaniam et al., 2011), and phosphorylation at this site is critical for ligand-dependent inhibition of trans-endothelial migration controlled by EphA2 (Locard-Paulet et al., 2016). To evaluate the specificity of the action of TYPE7 on EphA2, we performed control experiments with pHLIP. This peptide displays a similar pH-dependent membrane insertion to TYPE7 (Hunt et al., 1997; Scott et al., 2017), and has a similar content of acidic residues (Barrera et al., 2011; Fendos et al., 2013), but pHLIP displays low sequence homology with TYPE7 (Figure 3—figure supplement 2A). Specifically, we evaluated if EphA2 phosphorylation at Y772 or cell migration were affected by the membrane insertion of pHLIP. We observed that the presence of pHLIP changed neither EphA2 Y772 phosphorylation (Figure 3—figure supplement 2) nor cell migration (Figure 3—figure supplement 3), suggesting that the effect of TYPE7 is specific.

Intriguingly, TYPE7 and EA1 caused different JMS phosphorylation, as TYPE7 did not promote phosphorylation of Y588 and Y594 (Figure 3B,D,E). Additionally, we observed that TYPE7 did not induce cell proliferation or phosphorylation of S897 (Figure 3—figure supplement 4A–C), a residue phosphorylated by Akt, RSK, and PKA that promotes ligand-independent cell migration and invasion (Miao et al., 2009; Wang, 2011; Zhou et al., 2015; Barquilla et al., 2016). TYPE7 did not cause EphA2 expression changes either (Figure 3—figure supplement 4D–E). Last, we examined the specificity of TYPE7 using an array of 49 human RTK. The array data suggests that TYPE7 does not significantly increase tyrosine phosphorylation of other RTKs (Figure 3—figure supplement 5). Taken together, these results suggest that TYPE7 inhibits cell migration by inducing specific EphA2 phosphorylation at Y772, but not at the JMS.

The activation of EphA2 by ephrins elicits downstream signaling that inhibits cell migration (Shi and Wang, 2018). Akt is an important downstream target of EphA2 (49). When Akt is activated, it is phosphorylated at residues T308 and S473. Activation of EphA2 by ephrinA1 inhibits Akt and reduces phosphorylation at the two sites (Barquilla et al., 2016). We evaluated the effect of TYPE7 on the phosphorylation of Akt. We observed that TYPE7 significantly reduced phosphorylation at T308 and S473 to a degree that is comparable to the effect of EA1 (Figure 3F–H). Again, no changes were observed when pHLIP was used as a negative control.

### TYPE7 promotes limited self-assembly of EphA2

**Figure 4.**
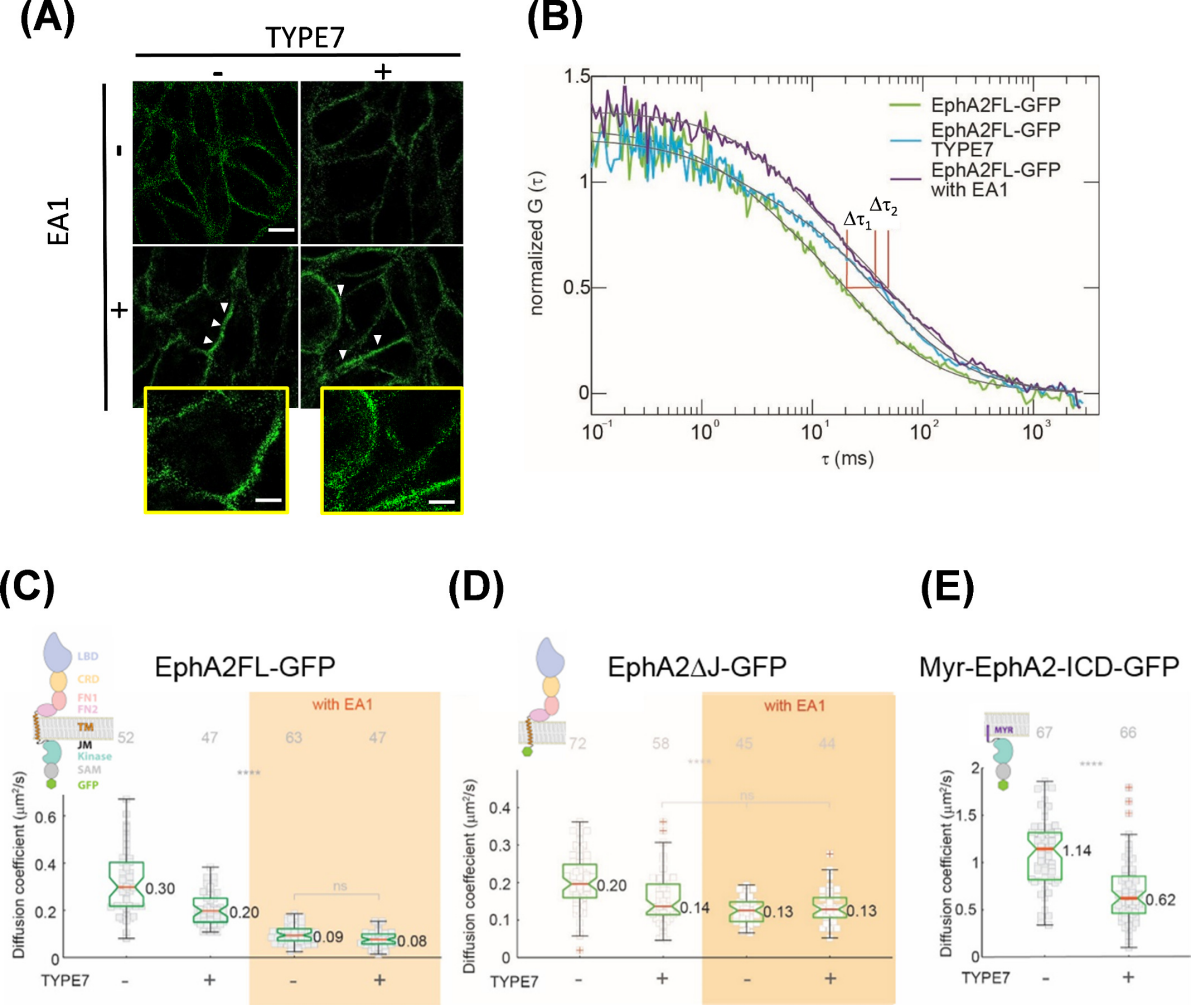
TYPE7 induces formation of oligomers of EphA2. (**A**) Super-resolution SIM data. H358 cells were incubated in the presence (+) or absence (-) of 0.5 µg/mL EA1 and 2 µM TYPE7. Representative images show fluorescence obtained using an anti-EphA2 antibody (*n* = 4). Scale bar is 10 µm. Insets magnify areas with clusters, and the scale bars are 5 µm. (**B**) Representative FCS autocorrelation curves for EphA2FL-GFP in control conditions (green) or in the presence of TYPE7 (blue) and EA1 (magenta). Δτ1 and Δτ2 represent the changes in dwell time. (**C–E**) Diffusion coefficient results, containing graphic models describing the EphA2 constructs used. (**C**) Box-whisker plot of measurement of the FCS diffusion coefficient of EphA2FL-GFP. (**D**) Diffusion coefficient of EphA2ΔJ-GFP. (**E**) Diffusion coefficient of Myr-EphA2 ICD-GFP. Diffusion coefficients collected from cells with and without TYPE7 treatment are reported along with EA1 ligand stimulation (orange boxes). The median values are reported next to the box plots. Each data point is the average of five 10 s FCS measurements on one cell. The grey numbers on top of the plots are the total number of cells measured. Criteria for the box, median, quartiles, whiskers and outliers are described elsewhere (Shi et al., 2017). One-way ANOVA tests were performed to obtain the *p* values (****p<0.0001; ns, not significant).

In the absence of ligand, EphA2 is found in a monomer-dimer equilibrium (Singh et al., 2015). However, differently to other receptor tyrosine kinases, EphA2 dimerization does not cause full receptor activation (Nikolov et al., 2014; Janes et al., 2012). Instead, stronger EphA2 activation is achieved upon dimer self-assembly into higher-order clusters that form extended signaling arrays. These clusters can contain hundreds of EphA2 molecules (Himanen et al., 2010; Nikolov et al., 2014; Janes et al., 2012) and appear as micron-sized puncta in the plasma membrane (Salaita et al., 2010). We explored if TYPE7 activates EphA2 by promoting receptor clustering. First, we employed super-resolution Structured Illumination Microscopy (SIM) to qualitatively investigate this possibility. Figure 4A shows that untreated cells have a relatively homogeneous EphA2 distribution at the plasma membrane. EA1 treatment caused EphA2 to concentrate in brighter foci on the membrane, indicating clusters of EphA2 (marked as white arrowheads). Strikingly, incubation with TYPE7 did not promote foci formation. Similar conclusions were drawn from confocal imaging (see Figure 2). This was surprising, since TYPE7 increased Y772 phosphorylation and reduced cell migration as effectively as EA1, but apparently, it did so without promoting formation of EphA2 foci. This suggests that TYPE7 and EA1 might achieve similar inhibition of cell migration despite inducing different levels of EphA2 self-assembly.

**Figure 4—figure supplement 1.**
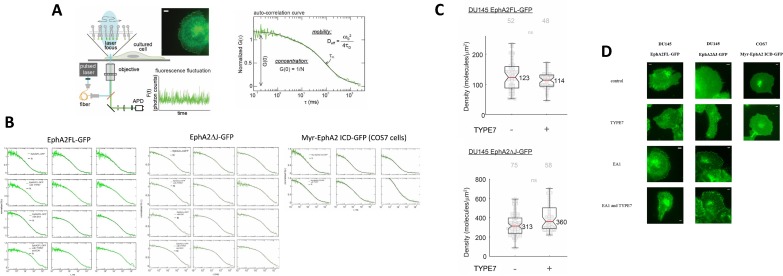
FCS supplement. (**A**) FCS experiments. Schematic diagram of a FCS experiment. A 488 nm laser beam is focused at the peripheral membrane area of a cultured cell to excite the GFP tag on the diffusive receptors. The emitted photons are collected through the objective and directed to an avalanche photodiode (APD). The fluorescence fluctuation caused by the diffusion of receptors is recorded and transformed into the auto-correlation function. Insert: epi-fluorescence image of DU145 cell expressing GFP-tagged receptors; the red dot represents the position of laser beam. Scale bar is 5 μm. In the auto-correlation curve, τ_D_ and G(0) report on the mobility and the concentration of the diffusive receptors, respectively. (**B**) FCS auto-correlation curves for the three EphA2 constructs. Three curves are shown for each experimental condition. (**C**) Receptor density of EphA2FL-GFP in DU145 cell membranes. Median density value is reported for EphA2FL-GFP and EphA2ΔJ-GFP. Each data point is the average of five 10 s FCS measurements on one cell. 52 cells were measured. (**D**) Representative epi-fluorescence images of cells used for FCS measurements under different conditions of TYPE7 and EA1 treatment. Scale bars are 5 μm.

**Figure 4—figure supplement 2.**
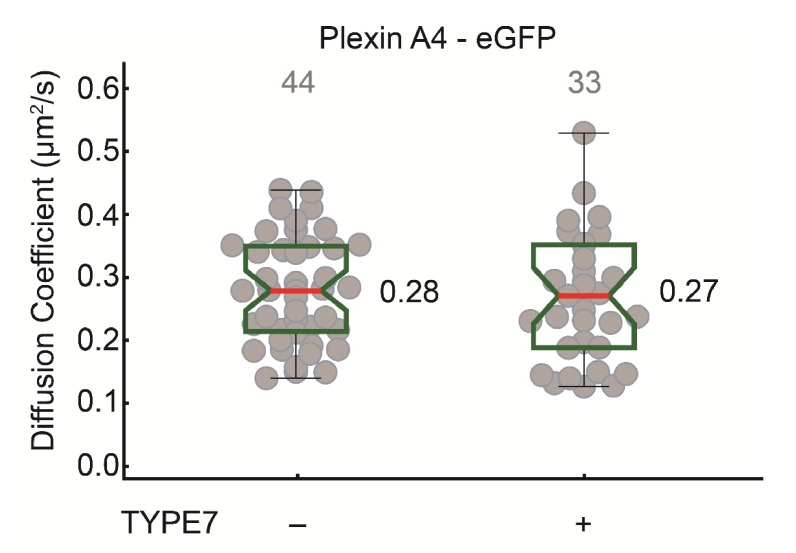
TYPE7 does not affect diffusion of PlexinA4, another single-pass transmembrane receptor. Box-whisker plot of measurement of the FCS diffusion coefficient of Plexin A4-eGFP wild type in COS-7 cells before and after TYPE7 stimulation.

**Figure 4—figure supplement 3.**
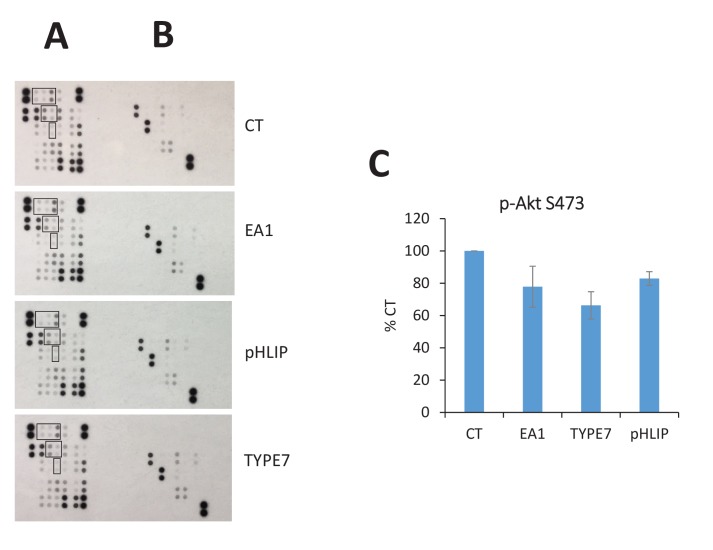
Human phospho-kinase array studies of TYPE7 specificity. H358 cells were treated for 10 min with TYPE7 (2 μM) and the following controls: Fc (CT), EA1 (0.5 μg/mL) and pHLIP (2 μM). After treatment, cell lysates were incubated overnight with array membranes (R and D Systems ARY003B) for duplicated detection of phosphorylation of 43 total kinases (**A**) and their substrates (**B**). Myristoylated Src family kinases are boxed: top (Hck, Fyn and Src), middle (Yes and Lyn), and bottom (Lck). The pHLIP peptide was used as a control for specificity. The array contains the following proteins, in order from top to bottom, and then left to right: p38α, ERK1/2, JNK 1/2/3, GSK-3α/β, p53, EGFR, MSK1/2, AMPKα1, Akt, p53, TOR, CREB, HSP27, AMPKα2, β-Catenin, p70 S6 Kinase, p53, c-Jun, Src, Lyn, Lck, STAT2, STAT5a, p70 S6 Kinase, RSK1/2/3, eNOS, Fyn, Yes, Fgr, STAT6, STAT5b, STAT3, p27, PLC-g1, Hck, Chk-2, FAX, PDGFRb, STAT5a/b, STAT3, WNK1, PYK2, PRAS40 and HSP60. (**C**) Quantification of Akt phosphorylation (p–S473).

To confirm these results, we used fluorescence correlation spectroscopy (FCS) to study EphA2 lateral organization in live cells. FCS is more sensitive than SIM for detecting changes in oligomerization status, particularly for small oligomers. FCS records the time-resolved fluorescence fluctuations within a confocal detection volume caused by diffusion of EphA2. By performing correlation analysis on the recorded fluctuation signals, auto-correlation function (ACF) curves are obtained (Figure 4—figure supplement 1A–B). From the ACF curve, we determined the lateral mobility of EphA2, reported as an effective diffusion coefficient (D). We investigated changes in EphA2 oligomeric state monitoring lateral mobility after TYPE7 and EA1 treatment. Although it is difficult to use lateral mobility to calculate the absolute size of EphA2 oligomers, there is a direct correlation between lateral mobility and oligomer size (Shi et al., 2017; Chung et al., 2010). Namely, for the same receptors in the same membrane environment, a decrease in the lateral mobility indicates growth in oligomer size. FCS measurements were recorded in live DU-145 cells (Figure 4B, Figure 4—figure supplement 1D) that stably express EphA2 labelled with enhanced GFP (EphA2FL-GFP) (Shi et al., 2017). While this experimental setting does not allow ruling out the presence of more than one diffusing component, a single relaxation term (Equation 5) fitted the data well. In untreated cells, the median D value for EphA2FL-GFP was 0.30 μm^2^/s (Figure 4C, first column). When treated with EA1, the median D value decreased to 0.09 μm^2^/s (Figure 4C, orange area). The decrease in D upon EA1 stimulation showed that, as expected, EphA2FL-GFP formed clusters (Shi et al., 2017). However, upon treatment with TYPE7, D decreased to 0.20 μm^2^/s (Figure 4C, second column). This indicates that EphA2FL-GFP oligomerizes upon TYPE7 treatment, but the intermediate D value indicates that EphA2 is detected in a lower-order oligomeric state than the cluster. The difference between diffusion coefficients obtained for EA1-activated EphA2FL-GFP, both with and without TYPE7, was not statistically significant. This suggests that regardless of the presence of TYPE7, EA1 caused EphA2FL-GFP to form clusters of similar size. This apparent saturation effect agrees with the cell migration and phosphorylation data (Figure 3A,D and E). Additionally, FCS data analysis allows us to quantify the plasma membrane levels of EphA2FL-GFP. Figure 4—figure supplement 1C shows that incubation with TYPE7 did not alter the levels of EphA2 expression, in agreement with Western blot data shown in Figure 3—figure supplement 4D–E.

To demonstrate that TYPE7 is specifically targeting EphA2 without affecting other single-pass transmembrane receptors, we tested the effect of TYPE7 on Plexin A4. Plexin A4 is a cell surface protein that has a similar domain structure as EphA2: one transmembrane domain, a large ectodomain, and an enzymatic cytoplasmic domain. Previous work showed that Plexin A4 forms an inactive dimer prior to ligand stimulation (Marita et al., 2015). COS-7 cells were transiently transfected with Plexin A4 labelled with eGFP (Plexin A4-eGFP). FCS measurements were carried out on the peripheral membrane area of live cells expressing Plexin A4-eGFP to measure any change in their lateral mobility upon TYPE7 treatment. In untreated cells, the median diffusion coefficient (D) value for Plexin A4-eGFP was 0.28 μm^2^/s (Figure 4—figure supplement 2), similar to the previously published value (Marita et al., 2015). There was no significant difference when the cells were treated with TYPE7 (D = 0.27 μm^2^/s). This control experiment suggests that TYPE7 does not affect the diffusion of transmembrane proteins in a non-specific manner.

The results obtained in lipid vesicles containing TMJM_563_-EphA2 suggested that TYPE7 interacts with the membrane-proximal region of EphA2. However, TMJM_563_-EphA2 encompasses not only the TM helix of EphA2 but also the first five basic JMS residues (Figure 1A). In order to define the domains of EphA2 that interact with TYPE7, we performed additional FCS experiments with two deletion EphA2 constructs. We first used a truncation construct where the full ICD was deleted at the first JMS residue (Figure 4D). The resulting construct, EphA2ΔJ-GFP (Shi et al., 2017), was used to study the ability of TYPE7 to target the EphA2 TM helix. Using this construct, we observed that TYPE7 treatment decreased the mobility of EphA2ΔJ-GFP from 0.20 μm^2^/s to 0.14 μm^2^/s, suggesting that TYPE7 binding to the TM domain increased oligomerization. Interestingly, upon EA1 stimulation, D was 0.13 μm^2^/s (Figure 4C, orange area), similar to the value observed with TYPE7. This suggests that in the absence of the ICD, TYPE7 has a similar effect as EA1 on self-assembly, suggesting that the ICD domains might be responsible for the differences in clustering observed between EA1 and TYPE7.

Finally, we studied the oligomerization of the isolated EphA2 ICD. FCS was thus performed using Myr-EphA2 ICD-GFP transfected in COS-7 cells. In Myr-EphA2 ICD-GFP, the ICD of EphA2 is anchored to the membrane at the first JMS residue using a myristoyl group (Shi et al., 2017). When we performed FCS experiments with this construct, we observed faster diffusion compared to the other two EphA2 construct in control conditions. Interestingly, treatment with TYPE7 also decreased D (Figure 4E), indicating that TYPE7 also promoted oligomerization of the ICD (cell images are shown at Figure 4—figure supplement 1D). We performed control experiments to evaluate if the oligomerization change that TYPE7 induces in Myr-EphA2 ICD-GFP might result from nonspecific interactions with the myristoyl moiety. To this end, we assayed the effect of TYPE7 on six Src family kinases, which are also linked to the membrane *via* myristoylation. Such experiments showed that TYPE7 did not alter the phosphorylation status of any of the myristoylated kinases (Figure 4—figure supplement 3). Additionally, we assayed the phosphorylation status of 37 other protein kinases and kinase substrates, with the exception of Akt (Figure 4—figure supplement 3C). Importantly, we observed that TYPE7 did not induce phosphorylation changes in any of these proteins. These results additionally suggest that the effects of TYPE7 on cell migration results from changes in EphA2 activity, and not any of these other cellular targets (Figure 4—figure supplement 3). Collectively, our data indicate that TYPE7 interacts with both the TM helix and the ICD of EphA2, to promote receptor oligomerization.

## Discussion

In this work, we show how strategic addition of acidic residues can transform a transmembrane domain into a water-soluble species, which can be triggered to insert into membranes. This finding can have important implications for the design of new ligands that modulate protein-protein interactions in membrane proteins. A molecule capable of establishing protein-protein interactions efficiently in cellular membranes should have three fundamental properties (Stone and Deber, 2017): (1) be easily deliverable into the membrane, where it should reside stably; (2) adopt an appropriate conformation to bind to the target; and (3) do not cause membrane disruption. Our data indicate that TYPE7 satisfies all these criteria. TYPE7 displays affinity for lipid bilayers, while it is readily soluble in buffer, which allows for easy plasma membrane delivery in physiological conditions. We hypothesize membrane binding of TYPE7 is initially driven by its moderately hydrophobic nature, as in the ATRAM and pHLIP peptides (Reshetnyak et al., 2007; Hunt et al., 1997; Deacon et al., 2015). After localizing at the surface of lipid vesicles, TYPE7 adopts a TM configuration, triggered by a pH decrease.

Our studies in cells, including co-precipitation (Figure 2), indicated that TYPE7 interacts with endogenous EphA2. Additional experiments in a reconstituted vesicle system showed that the acidity required for TYPE7 insertion significantly diminished in the presence of the membrane region of EphA2. In fact, in the presence of TMJM_563_-EphA2, the pH_50_ of TYPE7 membrane insertion shifted to a less acidic value, and the transition started at neutral pH (Figure 1D). No changes in pH_50_ were observed using a control transmembrane domain of different sequence, indicating that the interaction is specific. We propose that binding to TMJM_563_-EphA2 shifts the membrane equilibrium of TYPE7 away from the membrane surface, and promotes glutamic acid protonation and formation of the transmembrane state (Figure 1F).

The data obtained with TMJM_563_-EphA2 suggest an interaction between TYPE7 and the hydrophobic amino acids of the transmembrane helix of EphA2. However, the TMJM_563_-EphA2 peptide contains at the C-terminus a basic stretch, ^559^HRRRK^563^, corresponding to the start of the JMS. It has not escaped our notice that TYPE7 contains a potentially complementary acidic stretch at the C-terminus, with sequence EEEEE (Figure 1A), which might establish an attractive electrostatic interaction with the basic stretch of TMJM_563_-EphA2. We performed additional experiments to determine if TYPE7 could interact with the JMS of EphA2 in cells. Indeed, we observed that TYPE7 promoted self-assembly of the full ICD, containing the JMS but not the TM domain, as determined by FCS (Figure 4E). As expected, TYPE7 also promoted self-assembly of the EphA2 construct lacking the full JMS, but containing the TM domain (Figure 4D). Taken together our data suggest that TYPE7 interacts with EphA2 both at the TM domain and the ICD, and we hypothesize the ICD interaction occurs at the JMS.

We studied the biological effect of the interaction of TYPE7 with EphA2 using a trans-well migration assay. Interestingly, we observed that TYPE7 inhibited EphA2-driven cell migration to a similar extent as the saturating EA1 concentrations employed (Figure 3A). It has been shown that phosphorylation of the activation loop residue Y772 of EphA2 is required for ligand-induced inhibition of cell migration (Locard-Paulet et al., 2016; Singh et al., 2015). To determine the molecular mechanism of the activation of EphA2 by TYPE7, we studied the phosphorylation at the JMS and kinase activation loop. We observed that EA1 and TYPE7 caused a similar increase in Y772 phosphorylation, indicating that this molecular event might explain the similar effect of both ligands on cell migration.

Surprisingly, clear differences existed in the phosphorylation of the JMS residues Y588 and Y594. While TYPE7 did not affect their state, EA1 strongly promoted phosphorylation of Y588 and Y594. JMS phosphorylation is required for EA1 activation of EphA2, since the JMS auto-inhibits the kinase domain (Lemmon and Schlessinger, 2010). This regulatory mechanism involves docking of the JMS to the kinase domain, which stabilizes the inactive kinase state. EphA2 activation by ephrin binding promotes phosphorylation of the JMS residues Y588 and Y594, which causes a conformational change in the JMS that leads to its release from the kinase domain, and ends auto-inhibition (Wybenga-Groot et al., 2001). As a result, Y772 in the kinase activation loop is phosphorylated and the kinase domain is activated (Singh et al., 2015; Fang et al., 2008). Our results show that TYPE7 promotes full EphA2 Y772 phosphorylation and inhibition of cell migration without JMS phosphorylation. This suggests that phosphorylation of the JMS is not the only path to release juxtamembrane inhibition of EphA2. How can TYPE7 release the auto-inhibition without JMS phosphorylation? We hypothesize that the interaction between TYPE7 and the JMS of EphA2 might induce a conformational change that reorients the JMS without requiring phosphorylation, and as a result preclude autoinhibition by binding of this segment to the kinase domain. Interestingly, it has been recently reported that the regulation of the phosphorylation of Y772 and Y588 can be uncoupled by differential de-phosphorylation (Locard-Paulet et al., 2016). Our data indicates that phosphorylation of Y772 can occur *via* a different mechanism that does not require JMS phosphorylation. Our results illustrate the flexibility of molecular events involved in the interplay between the JMS and kinase domain, and might suggest that additional modes of release of autoinhibition could regulate EphA2 phosphorylation.

Crosstalk between Akt and EphA2 has been documented in several studies (Shi and Wang, 2018; Miao et al., 2009; Yang et al., 2011). Akt is a key protein that controls cell migration and differentiation through the oncogenic Akt/mTORC1 pathway (Altomare and Khaled, 2012). EphA2 activation by ephrinA1 downregulates this pathway through Akt de-phosphorylation mediated by a serine/threonine phosphatase (Yang et al., 2011). Figure 3F–H shows that TYPE7 decreased phosphorylation at the two main Akt kinase activation sites, T308 and S473, similarly to EA1. We propose that inhibition of Akt by TYPE7 can explain the strong inhibition of cell migration shown in Figure 3A. Furthermore, this observation suggests that TYPE7 can be used to inhibit the oncogenic Akt/mTORC1 signaling pathway.

**Figure 5.**
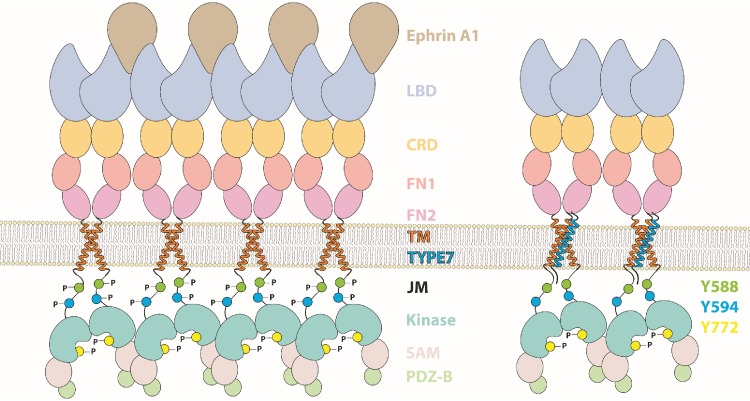
Cartoon depicting the different domains forming EphA2, which compares the activation mechanism of ephrinA1 (*left*) with the proposed TYPE7 mechanism (*right*), where the JMS is not phosphorylated and EphA2 assembles into smaller oligomers. Figure is not to scale.

EphA2 ligand-dependent activation involves formation of large clusters. We compared the effect of EA1 and TYPE7 on clustering. The FCS and SIM data in Figure 4 show that while EA1 promotes formation of large clusters of the full-length EphA2, TYPE7 does not induce clusters, but smaller oligomers. This indicates the possibility that the large EphA2 clusters that EA1 induces are not required for EphA2-mediated inhibition of cell migration. Based on this result, we suggest that a smaller oligomer might be the active signaling state of EphA2 (Figure 5). A similar scenario has been proposed for EphB2 using chemical dimerizers (Schaupp et al., 2014). The larger EphA2 clusters might be needed instead for regulation or recycling, as a means to control the duration and intensity of EphA2 signaling (Boissier et al., 2013).

It has been previously shown that TM peptides can modulate other membrane receptors. However, previous efforts typically involved expressing hydrophobic peptides in cells (Talbert-Slagle et al., 2009; Heim et al., 2015), or delivering peptides solubilized using detergents and/or organic solvents (Stone and Deber, 2017; Shandler et al., 2011; Lee et al., 2014; Arpel et al., 2014), which can be deleterious to cells, and incompatible with clinical applications. Our work represents a significant advance over those efforts, since TYPE7 targets cells in physiological conditions. Furthermore, the pH-dependent membrane insertion could potentially confer a means for the targeted delivery of TYPE7 to cells in acidic environments, such as tumors.

EphA2 is a promising target for therapeutics of different cancer types. Overexpression of EphA2 in cancer can promote cancer progression and malignancy, and it is often associated with ephrin downregulation (Macrae et al., 2005). Importantly, TYPE7 can activate EphA2 in the absence of ephrins. Furthermore, it has been proposed that EphA2 monomers are pro-tumorigenic (Singh et al., 2015). As TYPE7 promotes oligomerization, we hypothesize it might have an anti-tumorigenic effect. Moreover, TYPE7 inhibits cell migration without showing toxicity, making this peptide an interesting lead compound to reduce migration of cancerous cells and metastasis. Importantly, the strategy we have developed to target EphA2 can be generalized to design peptide tools to study the activation mechanism of other single-span and multi-span membrane receptors.

## Materials and methods

### Reagents and peptides

Peptides (TYPE7 and TMJM_563_-EphA2) were synthesized by Thermo Fisher Scientific (Waltham, MA) at ≥95% purity. Peptide purity was confirmed by matrix-assisted laser desorption ionization-time-of-flight (MALDI-TOF) mass spectrometry and high performance liquid chromatography (HPLC). The matrix α-cyano-4-hydroxycinnamic acid (α-HCCA) and trifluoroacetic acid (TFA) were purchased from Sigma-Aldrich (St. Louis, MO). Sodium phosphate and sodium acetate buffers were also purchased from Sigma-Aldrich (St. Louis, MO). HPLC-grade water and methanol were purchased from Fisher Chemical (Waltham, MA). Succinimidyl 6-(N-(7-nitrobenz-2-oxa-1,3-diazol-4-yl)amino) hexanoate (NBD-X, SE) was purchased from AnaSpec, Inc (Fremont, California). BODIPY FL-X SE, Alexa Fluor 568 SE, and DyLight 680 maleimide were purchased from Thermo-Fisher Scientific (Waltham, MA). Anti-EphA2 polyclonal antibody (EphA2 D4A2 XP), phospho-EphA2 (Y588-D7 × 2L), phospho-EphA2 (Y594), phospho-EphA2 (Y772), phospho-EphA2 (Y897-D9A1) and EphA2 (8B6) mouse antibody, Akt pan, phospho-Akt T308 and phospho-Akt S473 were purchased from Cell Signaling Technology (Danvers, MA). The anti-β-actin antibody was purchased from Abcam (Cambridge, MA).

### MALDI-TOF

Peptides were added to a saturated solution of α-HCCA in 70% methanol with 0.05% TFA. The resulting solution was dried onto the MSP AnchorChip target plate (Bruker, Billerica, MA) using the dried droplet method (Karas and Hillenkamp, 1988). The Bruker Microflex MALDI-TOF mass spetrometer was calibrated with the Bruker Peptide Calibration Standard II (Billerica, MA). Mass spectra were analyzed using FlexAnalysis software (Bruker, Billerica, MA).

### HPLC

To check purity, analytes (peptides, peptide-dye conjugates) were dissolved in methanol and injected into a semi-preparative Agilent Zorbax 300 SB-C18 column on an Agilent 1200 series HPLC system (Santa Clara, CA). The gradient from solvent A (water +0.05% TFA) to solvent B (methanol +0.05% TFA) was 50 min from 5% B to 100% B. Peptides typically eluted near 95 – 100% B.

### Peptide conjugation

TYPE7 was labeled at the N-terminus with NBD-X SE, DyLight 680 maleimide, and BODIPY FL-X SE. Unreacted dye was removed using HPLC or gel filtration through a PD-10 column (Life Technologies, Waltham, Massachusetts), and MALDI-TOF was used to determine that a single dye molecule was bound per peptide molecule with α-HCCA matrix.

### Liposome preparation

Lipids were purchased from Avanti Polar Lipids, Alabaster, AL. POPC (1-palmitoyl-2-oleoyl-sn-glycero-3-phosphocholine) and 22:1-PC (1,2-dierucoyl-sn-glycero-3-phosphocholine) stocks were prepared in chloroform. Aliquots of lipids were dried under a steady stream of argon gas and then placed in a vacuum overnight. The lipid films were resuspended with 10 mM sodium phosphate buffer (pH 7.9) and were then extruded with a Mini-Extruder (Avanti Polar Lipids, Alabaster, AL) through a 100 nm pore size membrane (Whatman, United Kingdom) to form large unilamellar vesicles (LUVs).

### Circular dichroism (CD)

The sample was prepared by incubation of TYPE7 with POPC LUVs, for a lipid to peptide molar ratio of 200:1. To reach the desired experimental pH, the pH of the samples was adjusted with the addition of either 100 mM sodium phosphate pH 8 or 100 mM sodium acetate pH 4. CD spectra were recorded on a Jasco J-815 spectropolarimeter at room temperature. For the solubility study, peptide samples were prepared in either PBS (pH 7.4) or 10 mM NaP_i_ pH 8 with a final concentration of 5 μM or 50 μM. The appropriate buffer backgrounds were subtracted.

### pH_50_ determination assay

TMJM_563_-EphA2 and GWALP23 stocks were prepared in trifluoroethanol. Dried films of POPC, POPC:TMJM_563_-EphA2 (molar ratio of 500:1), and POPC:GWALP23 (molar ratio of 500:1) were resuspended in 1 mM NaP_i_ pH 8. The POPC liposomes and proteo-liposomes were prepared via extrusion using a Mini Extruder to form ~100 nm large unilamellar vesicles. Lyophilized TYPE7 conjugated with NBD-X FL was also rehydrated with 1 mM NaP_i_ pH 8 and was incubated with the liposomes and proteo-liposomes with a final concentration of 0.2 μM. The POPC:TYPE7 molar ratio was 2000:1. For the titrations, a series of 100 mM buffers (sodium acetate and sodium phosphate) were used to achieve the desired pH, while keeping the total buffer concentration constant. The final pH of each individual well was measured. Fluorescence spectra were recorded at 25°C with excitation at 470 nm and an emission range of 520 – 600 nm using a Cytation five imaging plate reader (Biotek Instruments, Winooski, VT). Appropriate lipid blanks were prepared at the lowest and highest pH. The specific blanks were averaged and subtracted accordingly. Data were analyzed by calculating the center of mass (CM) of the fluorescence spectrum using the following equation:

(1) $$CM=\sum_{1}^{n}I_{i}\lambda_{i}/\sum_{1}^{n}I_{i}$$

where *Ii* is the fluorescence intensity measured at a wavelength λ*i*. (Barrera et al., 2002; Royer and Scarlata, 2008). The fluorescence center of mass (*F*) values at different pH were fitted to determine the pH_50_, using Equation 2:

(2) $$F=\left(F_{A}\;+F_{B}\;10^{m\left(pH-pH_{50}\right)}\;\right)/\left(1+10^{m\left(pH-pH_{50}\right)}\right)$$

where *F_A_* is the acidic baseline, *F_B_* is the basic baseline, *m* is the slope of the transition, and *pH_50_* is the midpoint of the curve.

### Oriented circular dichroism (OCD)

Stocks of POPC, TYPE7 and TMJM_563_-EphA2 were prepared in chloroform, methanol and TFE, respectively. Appropriate aliquots of lipid and peptide (50:1 lipid to peptide molar ratio) were first dried with argon gas and then placed under vacuum overnight. The lipid-peptide film was resuspended with methanol and spread on two circular quartz slides (Hellma Analytics, Germany). To ensure complete methanol evaporation, the slides were placed in a vacuum for 24 hr. After allowing the solvent to evaporate, the samples were hydrated with 150 μL of 100 mM sodium acetate buffer pH 4 – 5 overnight in 96% relative humidity, to obtain supported bilayers. The hydrated slides were assembled into the OCD cell, which had its inner cavity filled with saturated K_2_SO_4_ to keep the samples humidified. The OCD spectra were averaged for eight different rotations at 45° angles of the cell and recorded on a Jasco J-815 spectropolarimeter at room temperature. Appropriate lipid backgrounds were subtracted.

### Partition coefficient determination

Lyophilized samples of TYPE7-NBD were rehydrated in 10 mM NaPi (pH 8) at a final concentration of 0.8 μM and incubated with increasing concentrations of POPC LUVs. Emission spectra were recorded on a BioTek Cytation5 Cell Imaging Multi-Mode Reader. Three titration curves were averaged, and the resulting fluorescence intensity at 540 nm was plotted against the concentration of POPC in molar units. Fluorescence data (*F*) were fitted with OriginLab using:

(3) $$F=F_{0}\;+\Delta F\;\times \left(K_{p}x\right)/\left(55.3+K_{p}x\right)$$

where *F_0_* is the initial fluorescence intensity, *ΔF* is the change in fluorescence intensity, *x* is the lipid concentration, and 55.3 is the molar concentration of water. Equation 3 was used to determine the partition coefficient, *K*_p_, defined as the ratio of concentrations of a compound in a mixture of two phases.

### Calcein leakage assay

POPC LUVs were prepared as described above, but the dried POPC lipid film was rehydrated with 50 mM calcein in 10 mM HEPES and 50 mM EDTA (pH 8). Free calcein was removed by gel filtration through a PD-10 column. TYPE7 was added to the calcein/LUVs suspensions at different concentrations to achieve final peptide:lipid molar ratios of 0.0025 – 0.5% and incubated for 30 min at room temperature. The calcein leakage was tracked by measuring fluorescence using a Synergy two microplate reader (BioTek, Winooski, VT) at an excitation wavelength of 485 nm and an emission wavelength of 528 nm. Complete calcein release was reached by adding 20% Triton X-100, and melittin was used as a control for a leakage-inducing peptide.

### Cell culture

H358, A375, DU-145 and COS-7 cells from ATCC (Manassas, VA) were cultured in a humidified incubator under 5% CO_2_ in RPMI (H358), DMEM (A375) and alpha-MEM (COS-7) media (Invitrogen, Carlsbad, CA) supplemented with 10% fetal bovine serum, 50 U/mL penicillin and 50 μg/ml streptomycin. Cells were incubated overnight in serum free medium in presence or absence of TYPE7 and treated the next day with recombinant IgG1 Fc (R and D Systems, Minneapolis, MN) as a control or 0.5 μg/mL of recombinant mouse EphrinA1-Fc chimera (EA1) (R and D Systems, Minneapolis, MN) for 5 or 10 min. Cell line authentication and mycoplasma-free certification was performed by ATCC for all cell lines.

### Cell proliferation assay (MTS)

H358 cell viability was measured using the CellTiter 96 Aqueous One Solution (Promega, Madison, WI) according to the manufacturer’s protocol, which uses the reagent MTS (3-(4,5-dimethylthiazol-2-yl)−5-(3-carboxymethoxyphenyl)−2-(4-sulfophenyl)−2H-tetrazolium, inner salt). Briefly, cells were seeded (2 × 10^3^ cells per well for proliferation and 5 × 10^4^ for toxicity) 2 days prior the experiments in a 96 well plate, and exposed to vehicle or TYPE7 at different concentrations (0.5 μM, 1 μM and 2 μM) and 3 μg/mL of Fc or EA1 and incubated 48 hr (toxicity) or 24 hr (proliferation). The MTS assay was performed in 100 μL of DMEM phenol red free medium (Invitrogen, Carlsbad, CA) in each well and 20 μL of the CellTiter solution was added to the samples, then the plate was placed in the 37°C incubator with 5% CO_2_ until it reached the desired color. The absorbance at 490 nm was measured in a plate reader (Synergy 2, Biotek). The results are representative of three independent experiments, performed in triplicate. Cell proliferation was expressed as the percentage of vehicle control.

### Co-localization analysis

A375 cells were plated at a seeding density of 1 × 10^4^ cells per well in a glass-bottom 8-well slide (Ibidi, Munich, Germany) coated with 50 µg/mL rat tail collagen I (Gibco, Waltham, MA). Cells were serum starved ON. In order to block the slide surface, samples were pre-treated with DMEM containing 2 µM unlabeled TYPE7 for 1 hr at 37°C. Samples were then treated with 0.5 µg/mL EphrinA1-Fc (R and D Systems, Minneapolis, MN) and/or with 0.2 µM of TYPE7-Alexa 568 in PBS containing 1 mM MgCl_2_ and 100 microM CaCl_2_ (PBS^++^) for 5 min at room temperature followed by a 2 min wash with PBS^++^ and immediately fixed in 4% PFA. After blocking and permeabilizing, samples were incubated with rabbit anti-EphA2 primary antibody followed by secondary antibody labelling with goat-anti rabbit IgG Alexa488 (Invitrogen Carlsbad, CA).

Cells were imaged on a confocal laser scanning microscope (Zeiss LSM 710) with 63x and 100x objectives using Zen2 blue edition software. The Pearson correlation coefficient, *r*, was determined using the ImageJ Co-localization Threshold plugin. The *r* value can range from −1 for perfect exclusion to +1 for perfect co-localization, and 0 corresponds to random localization. We calculated *r* for whole images to reduce biases associated to selecting ROIs. However, we expect *r* to be higher at the plasma membrane, since a population of EphA2 was internalized, while TYPE7 remained at the plasma membrane, precluding co-localization. A second factor that reduced the measured correlation was the heterogeneous expression of EphA2, since some cells have negligible receptor levels (i.e. see red cell in the lower-right corner of Figure 2A). Pearson correlation coefficients were compared using a Student’s *t*-test assuming unequal variance in IBM SPSS Statistics Software (version 24).

### Co-precipitation

H358 cells were incubated with lysis buffer containing 150 mM NaCl, 50 mM Tris-HCl, pH 7.4, 5 mM EDTA and 1% NP-40 with protease inhibitors and phosphatase inhibitors for 30 min at 4°C. The insoluble fraction was eliminated through centrifugation at 10,000 × g for 30 min at 4°C. After the centrifugation, the lysates were incubated with anti-EphA2 antibody and protein A conjugated to Sepharose (Pierce Chemical, Rockford, IL) for 8 hr at 4°C. To quantify the total amount of protein loaded, 20 μL of the lysates was saved. Beads were washed four times with lysis buffer. Proteins were eluted in SDS-PAGE sample buffer, separated by SDS-PAGE electrophoresis, and analyzed by Western blotting of 16.5% tricine gel to detect TYPE7-DL that was precipitated with endogenous EphA2. Equal amounts of immuno-precipitate were resolved on a 10% SDS-polyacrylamide gel, and then electrophoretically transferred to 0.45 μm nitrocellulose membranes (Bio-Rad, Hercules, CA). Total cell lysates were also subjected to immunoblot. Membranes were blocked with a milk solution (150 mM NaCl, 20 mM Tris-HCl, 5% milk (w/v), 0.1% Tween (v/v), pH 7.5) and successively probed with primary (diluted 1:1000) and IR-dye-conjugated secondary antibodies (diluted 1:10,000). Immunoreactive bands and TYPE7-DL were detected using an Odyssey Infrared Scanner (Li-Cor Biosciences, Lincoln, NE).

### Protein arrays

The human Proteome Profiler Phospho-RTK Array Kit, which covers 49 different RTKs in duplicate (catalog number ARY001B), and the 43-protein Proteome Profiler Human Phospho-Kinase Array Kit (catalog number ARY003B), were purchased from R and D systems. H358 cells were starved O.N and treated with Fc, 2 μM of TYPE7 or 0.5 μg/mL of EA1 for 10 min. The assay was performed accordingly to the manufacturer protocol. Briefly, H358 cells were lysed in the provided lysis buffer with protease and phosphatase inhibitors, then incubated overnight with the nitrocellulose membranes containing the immobilized RTK tested. The membranes were then incubated with the anti-Phospho-Tyrosine-HRP detection antibody and visualized with the kit Chemi Reagent Mix.

### Structure Illumination microscopy (SIM)

After the specific treatment, cells were fixed with 4% paraformaldehyde and subsequently permeabilized with PBS^++^ containing 1 mg/mL bovine serum albumin and 0.1% Triton X-100. Nonspecific binding was blocked using goat serum dilution buffer GSDB (33% goat serum, 40 mM NaPi, pH 7.4, 450 mM NaCl, and 0.6% Triton X-100). Anti-Epha2 rabbit primary and Alexa Fluor-conjugated secondary (Invitrogen, Carlsbad, CA) antibodies were diluted in GSDB and incubated for 1 hr at room temperature. Cells were visualized on a laser scanning microscope (model LSM 510; Carl Zeiss Microimaging, Thornwood, NY). Contrast and brightness settings were chosen so that all pixels were in the linear range. Images are the product of eightfold line averaging.

### Boyden chamber assay

1 × 10^5^ A375 or H358 cells were starved for 24 hr before the experiment, then treated in serum-free medium. Cells were seeded on the top chamber of polycarbonate 8 μm pore size membrane costar trans-well chambers (Corning Life Sciences, Corning, NY). EA1, Fc (1 μg/mL) or TYPE7 were added to the lower chamber together with 5% FBS. Cells were allowed to migrate for 24 hr after which the cells on top of the chamber were removed with a cotton swab, and the bottom chamber was fixed with 4% PFA. After staining with eosin and hematoxylin, the cells that passed through the filter and stayed on the undersides of inserts were counted under a bight field microscope with 20x objective. Images are representative of three independent experiments, with an average of 4 images per sample condition.

### FCS cell culture and plasmids

EphA2FL (residues 1 – 971) and EphA2ΔJ (residues 1 – 558) were amplified via PCR from human EphA2 cDNA, and cloned into pEGFP-C1 plasmid. The resulting EGFP fusion genes were inserted into a LZRS-Pac retrovirus vector and then transfected into Phoenix retroviral packaging cells for retrovirus production. DU145 cells were infected with retroviral-mediated gene transfer in the presence of 6 μg/mL polybrene and selected with 1 μg/mL of puromycin. The DU145 cells with stable EphA2 expression were cultured in a collagen-coated 10 cm dish with DMEM (10% FBS). EphA2 ICD (residues 559 – 971) was amplified via PCR from human EphA2 cDNA and cloned into pEGFP-N1 vector. The c-Src membrane localization sequence was inserted before the EphA2 gene. The resulting Myr-EphA2 ICD-GFP was transfected into COS-7 (ATCC, Manassas, VA) cells using Lipofectamine2000 (Invitrogen, Carlsbad, CA). COS-7 cells were cultured in a 10 cm dish with DMEM (10% FBS). All constructs lack the PDZ domain, as described elsewhere (Shi et al., 2017). Experiments with plexin A4 were performed as described elsewhere (Marita et al., 2015).

### FCS data collection

FCS measurements were performed with a customized inverted confocal fluorescence microscopy (Eclipse Ti, Nikon) equipped with a 100x TIRF objective (NA 1.47, oil, Nikon). The 488 nm excitation laser beam was separated from a continuum white light laser (9.7 MHz) (NKT Photonics, Denmark) using a narrow-band excitation filter (488: LL01-488-12.5) (Semrock, Rochester, New York). The beam was focused onto the live cell samples sitting in an on-stage incubator by the objective. The emission light from the sample was collected through the same objective and directed passing a 520/44 nm emission filter (FF01-520/44-25) (Semrock, Rochester, New York). The photons from the emission beam were collected by a single photon avalanche diode (SPAD) detector (Micro Photon Devices, Italy) and recorded with a time-correlated single photon counting (TCSPC) module (Picoharp 300, PicoQuant). Data was processed and analyzed with a Matlab script.

Excitation laser beam at 300 nW was focused on the live cells samples at 37°C. Laser was always parked at the edge of a flat membrane area where there was only homogenous fluorescence (Figure 4—figure supplement 1A). Five 15 s measurements were performed on one cell and were averaged and registered as one data point. Auto-correlation was performed on the recorded time-resolved fluorescence fluctuation traces (F(t)) according to the following equation:

(4) $$G\left(\tau \right)=\frac{\left\langle F(t+\tau )F(t)\right\rangle }{{\left\langle F(t)\right\rangle }^{2}}$$

where τ is the lag time, $G\left(\tau \right)$ is the auto-correlation function and ⟨ ⟩ stands for time average. The correlation of F(t) rendered auto-correlation function (ACF) curve was fitted with a diffusion model shown here:

(5) Gτ=1N1-F+Fe-τ/τT1-F11+ττD

where *N* is average number of fluorescent particles, $\tau_{D}$ is the average dwell time of fluorescent particles within the detection volume, *F* is the fraction of molecules in the triplet state, $\tau_{T}$ is the triplet relaxation time. The diffusion coefficient (*D*) was calculated based on $\tau_{D}$,

(6) $$D=\frac{\omega_{0}^{2}}{\tau_{D}}$$

where $\omega_{0}$ is the waist of the laser focus. The density was calculated by dividing *N* with the detection area that was calibrated with standard dye molecule with known diffusion coefficient.

### Statistical analysis

Unless indicated otherwise, data are reported as mean ± standard deviation (S.D), and resulted from three or more independent experiments. To evaluate differences between sample means, Student’s *t*-tests or ANOVA were performed. We used IBM SPSS (version 25) and Origin 9.1 to perform *t*-tests. For each *t*-test homogeneity was checked and the correct test assuming or not assuming equal variance was applied. The same software package was used for to perform the Mann-Whitney U test to the co-precipitation data. Statistical significance was considered as p<0.05. Where multiple comparisons were performed, significance was determined by *t*-tests followed by the Benjamini-Hochberg procedure using a false discovery rate of 0.05. Effect sizes in standard deviations were determined by Hedge’s *g* values as calculated in Excel 2016.

## Data Availability

Source data for the figures is included as Source data 1.

## Additional files

Source data 1.

Transparent reporting form

## References

[bib1] Altomare DA, Khaled AR (2012). Homeostasis and the importance for a balance between AKT/mTOR activity and intracellular signaling. Current medicinal chemistry.

[bib2] Anbazhagan V, Schneider D (2010). The membrane environment modulates self-association of the human GpA TM domain--implications for membrane protein folding and transmembrane signaling. Biochimica Et Biophysica Acta (BBA) - Biomembranes.

[bib3] Arpel A, Sawma P, Spenlé C, Fritz J, Meyer L, Garnier N, Velázquez-Quesada I, Hussenet T, Aci-Sèche S, Baumlin N, Genest M, Brasse D, Hubert P, Crémel G, Orend G, Laquerrière P, Bagnard D (2014). Transmembrane domain targeting peptide antagonizing ErbB2/Neu inhibits breast tumor growth and metastasis. Cell Reports.

[bib4] Balasubramaniam D, Paul LN, Homan KT, Hall MC, Stauffacher CV (2011). Specificity of HCPTP variants toward EphA2 tyrosines by quantitative selected reaction monitoring. Protein Science.

[bib5] Barquilla A, Pasquale EB (2015). Eph receptors and ephrins: therapeutic opportunities. Annual Review of Pharmacology and Toxicology.

[bib6] Barquilla A, Lamberto I, Noberini R, Heynen-Genel S, Brill LM, Pasquale EB (2016). Protein kinase A can block EphA2 receptor-mediated cell repulsion by increasing EphA2 S897 phosphorylation. Molecular Biology of the Cell.

[bib7] Barrera FN, Garzón MT, Gómez J, Neira JL (2002). Equilibrium unfolding of the C-terminal SAM domain of p73. Biochemistry.

[bib8] Barrera FN, Weerakkody D, Anderson M, Andreev OA, Reshetnyak YK, Engelman DM (2011). Roles of carboxyl groups in the transmembrane insertion of peptides. Journal of Molecular Biology.

[bib9] Binns KL, Taylor PP, Sicheri F, Pawson T, Holland SJ (2000). Phosphorylation of tyrosine residues in the kinase domain and juxtamembrane region regulates the biological and catalytic activities of Eph receptors. Molecular and Cellular Biology.

[bib10] Bocharov EV, Mayzel ML, Volynsky PE, Mineev KS, Tkach EN, Ermolyuk YS, Schulga AA, Efremov RG, Arseniev AS (2010). Left-handed dimer of EphA2 transmembrane domain: Helix packing diversity among receptor tyrosine kinases. Biophysical Journal.

[bib11] Boissier P, Chen J, Huynh-Do U (2013). EphA2 signaling following endocytosis: role of Tiam1. Traffic.

[bib12] Boyd AW, Bartlett PF, Lackmann M (2014). Therapeutic targeting of EPH receptors and their ligands. Nature Reviews Drug Discovery.

[bib13] Bürck J, Wadhwani P, Fanghänel S, Ulrich AS (2016). Oriented circular dichroism: a method to characterize Membrane-Active peptides in oriented lipid bilayers. Accounts of Chemical Research.

[bib14] Chung I, Akita R, Vandlen R, Toomre D, Schlessinger J, Mellman I (2010). Spatial control of EGF receptor activation by reversible dimerization on living cells. Nature.

[bib15] Davis S, Gale NW, Aldrich TH, Maisonpierre PC, Lhotak V, Pawson T, Goldfarb M, Yancopoulos GD (1994). Ligands for EPH-related receptor tyrosine kinases that require membrane attachment or clustering for activity. Science.

[bib16] Deacon JC, Engelman DM, Barrera FN (2015). Targeting acidity in diseased tissues: mechanism and applications of the membrane-inserting peptide, pHLIP. Archives of biochemistry and biophysics.

[bib17] Fang WB, Brantley-Sieders DM, Hwang Y, Ham AJ, Chen J (2008). Identification and functional analysis of phosphorylated tyrosine residues within EphA2 receptor tyrosine kinase. Journal of Biological Chemistry.

[bib18] Fendos J, Barrera FN, Engelman DM (2013). Aspartate embedding depth affects pHLIP's insertion pKa. Biochemistry.

[bib19] Heim EN, Marston JL, Federman RS, Edwards AP, Karabadzhak AG, Petti LM, Engelman DM, DiMaio D (2015). Biologically active LIL proteins built with minimal chemical diversity. PNAS.

[bib20] Himanen JP, Yermekbayeva L, Janes PW, Walker JR, Xu K, Atapattu L, Rajashankar KR, Mensinga A, Lackmann M, Nikolov DB, Dhe-Paganon S (2010). Architecture of Eph receptor clusters. PNAS.

[bib21] Hirai H, Maru Y, Hagiwara K, Nishida J, Takaku F (1987). A novel putative tyrosine kinase receptor encoded by the eph gene. Science.

[bib22] Holt A, Koehorst RB, Rutters-Meijneke T, Gelb MH, Rijkers DT, Hemminga MA, Killian JA (2009). Tilt and rotation angles of a transmembrane model peptide as studied by fluorescence spectroscopy. Biophysical Journal.

[bib23] Hunt JF, Rath P, Rothschild KJ, Engelman DM, Spontaneous EDM (1997). Spontaneous, pH-dependent membrane insertion of a transbilayer alpha-helix. Biochemistry.

[bib24] Janes PW, Nievergall E, Lackmann M (2012). Concepts and consequences of Eph receptor clustering. Seminars in Cell & Developmental Biology.

[bib25] Jun G, Guo H, Klein BE, Klein R, Wang JJ, Mitchell P, Miao H, Lee KE, Joshi T, Buck M, Chugha P, Bardenstein D, Klein AP, Bailey-Wilson JE, Gong X, Spector TD, Andrew T, Hammond CJ, Elston RC, Iyengar SK, Wang B (2009). EPHA2 is associated with age-related cortical cataract in mice and humans. PLOS Genetics.

[bib26] Kania A, Klein R (2016). Mechanisms of ephrin-Eph signalling in development, physiology and disease. Nature Reviews Molecular Cell Biology.

[bib27] Karas M, Hillenkamp F (1988). Laser desorption ionization of proteins with molecular masses exceeding 10,000 daltons. Analytical Chemistry.

[bib28] Killian JA, Nyholm TK (2006). Peptides in lipid bilayers: the power of simple models. Current Opinion in Structural Biology.

[bib29] Kucerka N, Nieh M-P, Katsaras J (1808). Fluid phase lipid Areas and bilayer thicknesses of commonly used phosphatidylcholines as a function of temperature. Biochimica Et Biophysica Acta.

[bib30] Kucerka N, Gallová J, Uhríková D, Balgavý P, Bulacu M, Marrink SJ, Katsaras J (2009). Areas of monounsaturated diacylphosphatidylcholines. Biophysical Journal.

[bib31] Kullander K, Klein R (2002). Mechanisms and functions of Eph and ephrin signalling. Nature Reviews Molecular Cell Biology.

[bib32] Lee J, Miyazaki M, Romeo GR, Shoelson SE (2014). Insulin receptor activation with transmembrane domain ligands. Journal of Biological Chemistry.

[bib33] Lemmon MA, Schlessinger J (2010). Cell signaling by receptor tyrosine kinases. Cell.

[bib34] Lisabeth EM, Falivelli G, Pasquale EB (2013). Eph receptor signaling and ephrins. Cold Spring Harbor Perspectives in Biology.

[bib35] Locard-Paulet M, Lim L, Veluscek G, McMahon K, Sinclair J, van Weverwijk A, Worboys JD, Yuan Y, Isacke CM, Jørgensen C (2016). Phosphoproteomic analysis of interacting tumor and endothelial cells identifies regulatory mechanisms of transendothelial migration. Science Signaling.

[bib36] Macrae M, Neve RM, Rodriguez-Viciana P, Haqq C, Yeh J, Chen C, Gray JW, McCormick F (2005). A conditional feedback loop regulates Ras activity through EphA2. Cancer Cell.

[bib37] Manders EM, Stap J, Brakenhoff GJ, van Driel R, Aten JA (1992). Dynamics of three-dimensional replication patterns during the S-phase, analysed by double labelling of DNA and confocal microscopy. Journal of Cell Science.

[bib38] Marita M, Wang Y, Kaliszewski MJ, Skinner KC, Comar WD, Shi X, Dasari P, Zhang X, Smith AW (2015). Class A plexins are organized as preformed inactive dimers on the cell surface. Biophysical Journal.

[bib39] Martinez-Outschoorn UE, Prisco M, Ertel A, Tsirigos A, Lin Z, Pavlides S, Wang C, Flomenberg N, Knudsen ES, Howell A, Pestell RG, Sotgia F, Lisanti MP (2011). Ketones and lactate increase cancer cell "stemness," driving recurrence, metastasis and poor clinical outcome in breast cancer: achieving personalized medicine via Metabolo-Genomics. Cell cycle.

[bib40] Miao H, Burnett E, Kinch M, Simon E, Wang B (2000). Activation of EphA2 kinase suppresses integrin function and causes focal-adhesion-kinase dephosphorylation. Nature Cell Biology.

[bib41] Miao H, Li DQ, Mukherjee A, Guo H, Petty A, Cutter J, Basilion JP, Sedor J, Wu J, Danielpour D, Sloan AE, Cohen ML, Wang B (2009). EphA2 mediates ligand-dependent inhibition and ligand-independent promotion of cell migration and invasion via a reciprocal regulatory loop with Akt. Cancer Cell.

[bib42] Nguyen VP, Alves DS, Scott HL, Davis FL, Barrera FN (2015). A Novel Soluble Peptide with pH-Responsive Membrane Insertion. Biochemistry.

[bib43] Nikolov DB, Xu K, Himanen JP (2014). Homotypic receptor-receptor interactions regulating Eph signaling. Cell Adhesion & Migration.

[bib44] Ozdirekcan S, Rijkers DT, Liskamp RM, Killian JA (2005). Influence of flanking residues on tilt and rotation angles of transmembrane peptides in lipid bilayers. A solid-state 2H NMR study. Biochemistry.

[bib45] Rankenberg JM, Vostrikov VV, Greathouse DV, Grant CV, Opella SJ, Koeppe RE (2012). Properties of membrane-incorporated WALP peptides that are anchored on only one end. Biochemistry.

[bib46] Reshetnyak YK, Segala M, Andreev OA, Engelman DM (2007). A monomeric membrane peptide that lives in three worlds: in solution, attached to, and inserted across lipid bilayers. Biophysical Journal.

[bib47] Rosenberger AF, Rozemuller AJ, van der Flier WM, Scheltens P, van der Vies SM, Hoozemans JJ (2014). Altered distribution of the EphA4 kinase in hippocampal brain tissue of patients with Alzheimer's disease correlates with pathology. Acta Neuropathologica Communications.

[bib48] Royer CA, Scarlata SF (2008). Fluorescence approaches to quantifying biomolecular interactions. Methods in Enzymology.

[bib49] Sabet O, Stockert R, Xouri G, Brüggemann Y, Stanoev A, Bastiaens PI (2015). Ubiquitination switches EphA2 vesicular traffic from a continuous safeguard to a finite signalling mode. Nature Communications.

[bib50] Salaita K, Nair PM, Petit RS, Neve RM, Das D, Gray JW, Groves JT (2010). Restriction of receptor movement alters cellular response: physical force sensing by EphA2. Science.

[bib51] Schaupp A, Sabet O, Dudanova I, Ponserre M, Bastiaens P, Klein R (2014). The composition of EphB2 clusters determines the strength in the cellular repulsion response. The Journal of Cell Biology.

[bib52] Schornack PA, Gillies RJ (2003). Contributions of cell metabolism and H+ diffusion to the acidic pH of tumors. Neoplasia.

[bib53] Scott HL, Westerfield JM, Barrera FN (2017). Determination of the Membrane Translocation pK of the pH-Low Insertion Peptide. Biophysical Journal.

[bib54] Shandler SJ, Korendovych IV, Moore DT, Smith-Dupont KB, Streu CN, Litvinov RI, Billings PC, Gai F, Bennett JS, DeGrado WF (2011). Computational design of a β-peptide that targets transmembrane helices. Journal of the American Chemical Society.

[bib55] Sharonov GV, Bocharov EV, Kolosov PM, Astapova MV, Arseniev AS, Feofanov AV (2014). Point mutations in dimerization motifs of the transmembrane domain stabilize active or inactive state of the EphA2 receptor tyrosine kinase. Journal of Biological Chemistry.

[bib56] Shi X, Hapiak V, Zheng J, Muller-Greven J, Bowman D, Lingerak R, Buck M, Wang BC, Smith AW (2017). A role of the SAM domain in EphA2 receptor activation. Scientific Reports.

[bib57] Shi X, Wang B (2018). Caught in the "Akt": Cross-talk between EphA2 and EGFR through the Akt-PIKfyve axis maintains cellular sensitivity to EGF. Science Signaling.

[bib58] Singh DR, Ahmed F, King C, Gupta N, Salotto M, Pasquale EB, Hristova K (2015). EphA2 Receptor Unliganded Dimers Suppress EphA2 Pro-tumorigenic Signaling. Journal of Biological Chemistry.

[bib59] Stone TA, Deber CM (2017). Therapeutic design of peptide modulators of protein-protein interactions in membranes. Biochimica Et Biophysica Acta (BBA) - Biomembranes.

[bib60] Talbert-Slagle K, Marlatt S, Barrera FN, Khurana E, Oates J, Gerstein M, Engelman DM, Dixon AM, Dimaio D (2009). Artificial transmembrane oncoproteins smaller than the bovine papillomavirus E5 protein redefine sequence requirements for activation of the platelet-derived growth factor beta receptor. Journal of Virology.

[bib61] Tandon M, Vemula SV, Mittal SK (2011). Emerging strategies for EphA2 receptor targeting for cancer therapeutics. Expert Opinion on Therapeutic Targets.

[bib62] Ulmschneider MB, Ulmschneider JP, Schiller N, Wallace BA, von Heijne G, White SH (2014). Spontaneous transmembrane helix insertion thermodynamically mimics translocon-guided insertion. Nature Communications.

[bib63] Wang B (2011). Cancer cells exploit the Eph-ephrin system to promote invasion and metastasis: tales of unwitting partners. Science Signaling.

[bib64] Wu Y, Huang HW, Olah GA (1990). Method of oriented circular dichroism. Biophysical Journal.

[bib65] Wybenga-Groot LE, Baskin B, Ong SH, Tong J, Pawson T, Sicheri F (2001). Structural basis for autoinhibition of the Ephb2 receptor tyrosine kinase by the unphosphorylated juxtamembrane region. Cell.

[bib66] Yang NY, Fernandez C, Richter M, Xiao Z, Valencia F, Tice DA, Pasquale EB (2011). Crosstalk of the EphA2 receptor with a serine/threonine phosphatase suppresses the Akt-mTORC1 pathway in cancer cells. Cellular Signalling.

[bib67] Zhou Y, Yamada N, Tanaka T, Hori T, Yokoyama S, Hayakawa Y, Yano S, Fukuoka J, Koizumi K, Saiki I, Sakurai H (2015). Crucial roles of RSK in cell motility by catalysing serine phosphorylation of EphA2. Nature Communications.

